# Validation of Twitter opinion trends with national polling aggregates: Hillary Clinton vs Donald Trump

**DOI:** 10.1038/s41598-018-26951-y

**Published:** 2018-06-06

**Authors:** Alexandre Bovet, Flaviano Morone, Hernán A. Makse

**Affiliations:** Levich Institute and Physics Department, City College of New York, New York, New York 10031 USA

## Abstract

Measuring and forecasting opinion trends from real-time social media is a long-standing goal of big-data analytics. Despite the large amount of work addressing this question, there has been no clear validation of online social media opinion trend with traditional surveys. Here we develop a method to infer the opinion of Twitter users by using a combination of statistical physics of complex networks and machine learning based on hashtags co-occurrence to build an in-domain training set of the order of a million tweets. We validate our method in the context of 2016 US Presidential Election by comparing the Twitter opinion trend with the New York Times National Polling Average, representing an aggregate of hundreds of independent traditional polls. The Twitter opinion trend follows the aggregated NYT polls with remarkable accuracy. We investigate the dynamics of the social network formed by the interactions among millions of Twitter supporters and infer the support of each user to the presidential candidates. Our analytics unleash the power of Twitter to uncover social trends from elections, brands to political movements, and at a fraction of the cost of traditional surveys.

## Introduction

Several works have shown the potential of online social media, in particular of the microblogging platform Twitter, for analyzing the public sentiment in general^1–4^ or to predict stock markets movements or sales performance^5–9^. With the increasing importance of Twitter in political discussions, a considerable number of studies^10–27^ also investigated the possibility to analyze political processes and predict political elections from data collected on Twitter. However, these initial investigations achieved only mixed results when compared to traditional surveys and engendered a number of critical studies^20,28,29^ questioning their methods and findings. One of the main criticisms is that instead of measuring the political support for a candidate or a party, they measure the political attention toward it, those two concepts being not necessarily correlated. Indeed, most work compare the volume of tweets, or mentions, related to the different candidates with traditional polls or election results. Lexicon-based sentiment analysis^30–32^ has also been used to improve this approach by attributing a positive or negative sentiment to the tweets containing mentions of the candidates or parties. However, not only does lexicon-based approach perform poorly on the informal, unstructured, sometimes ironic, language of Twitter^33^, but classifying sentiment as positive or negative does not allow one to differentiate simple attention from political support, especially during political scandals^29^. In this case, correctly capturing the context of the events is crucial to measure support.

Recent works^34,35^ have shown that by going beyond sentiment analysis, and by considering all the terms used in tweets, even the terms usually considered neutral, a more accurate measurement of the Twitter opinion during the 2012 US election was possible. Moreover, evidences suggest that it is possible to differentiate Republican and Democrat Twitter users based only on their usage of words^36^. Ceron *et al*.^34,37,38^ used a supervised machine learning approach based on a hand labeled training set to estimate the proportion of tweets in favor of each candidate in the 2012 US election and the 2012 Italian center-left primaries. Beauchamp^35^ extracted significant textual features from Twitter by fitting a model to existing polls and showed that these features improved state level polls prediction. Despite all these improvements, opinion time series derived from Twitter have not been validated so far with any traditional polling performed at the large scale.

Here, we are interested to capture the opinion trend in Twitter and compare it to opinion time series from independent off-line surveys. For this purpose, the setting of the 2016 US Presidential Election allows us to have access to a large aggregate of traditional surveys performed at regular intervals to compare with our Twitter opinion trend. We develop a supervised learning approach to measure the opinion of Twitter users where we do not try to classify tweets as expressing positive or negative sentiment, but as supporting or opposing one of two top candidates: Hillary Clinton and Donald Trump. Our approach innovates by using the network of hashtag co-occurrence to discover all the hashtags relevant to the elections and to assess the consistency of our hashtag classification. This allows us to automatically build a training set of the order of one million documents, which is two order of magnitude larger than what previous methods based on hand labeling^34,39^ typically allows. Moreover, using an in-domain training set not only helps us to capture the informalities of Twitter language, but also permits us to capture the rich context of the 2016 US election. We show that we can precisely measure the supports of each candidate in Twitter, and that while our approach is independent of traditional polls, the opinion trend we measure in Twitter closely matches the New York Times (NYT) National Polling Average^40^ and anticipates it by several days.

The agreement we find significantly exceeds results of previous attempts comparing Twitter-based metric time series with traditional polls time series^10,13,17,29,34^. We perform a systematic benchmark against previous methods and show that our method outperform all of them. The existence of a lag of several days between Twitter opinion and traditional surveys has already been noted^10,41^ by looking for the time shift giving the best correlations between Twitter opinion time series and traditional polls. Here, the existence of a 10 days time delay between Twitter opinion trend and the NYT National Polling Average is not only shown by a high correlation between the two time-series but also by the remarkable agreement of the fit of the two time-series. We also go beyond a post-hoc comparison of the Twitter and NYT opinion time series by showing that while training our model only on the first part of our dataset, our Twitter opinion trend is still in agreement with the results of the NYT polls during the last two months of the election period. We thus validate the use of Twitter activity to capture trends existing in the society at the national level. We also show that, contrary to our measure of the supports of each candidate, the attention toward the candidates, measured by previous studies^10,13,17,29^, does not agree with the NYT national polls.

By classifying at the tweet level and then at the user level we correctly take into account the difference of activity of supporters to extract the percentage of users in favor of each candidate contrary to methods that directly estimate the aggregated repartition of tweets^34,37,39^. This approach allows us to correctly interpret the Twitter opinion trend as the result of the variations in engagement of the supporters of each camp and to gain unique insight on the dynamics and structure of the social network of Twitter users in relation to their political opinion.

## Results

### Social network of Twitter users

We collect tweets mentioning the two top candidates in the 2016 US presidential election from June 1st until election day on November 8th, 2016 by using Twitter Search API to retrieve the following queries: *trump OR realdonaldtrump OR donaldtrump* and *hillary OR clinton OR hillaryclinton*. The resulting dataset consists of 98 million tweets with the keywords about Donald Trump sent by 6.7 million users and 78 million tweets with the keywords about Hillary Clinton sent by 8.8 million users. The combination of the two datasets results in a total of 171 million unique tweets. The total number of users is 11 million with an average of 1.1 million tweets per day (standard deviation of 0.6 million) sent by an average of about 375,000 distinct users (standard deviation of 190,000) per day.

**Figure 1:**
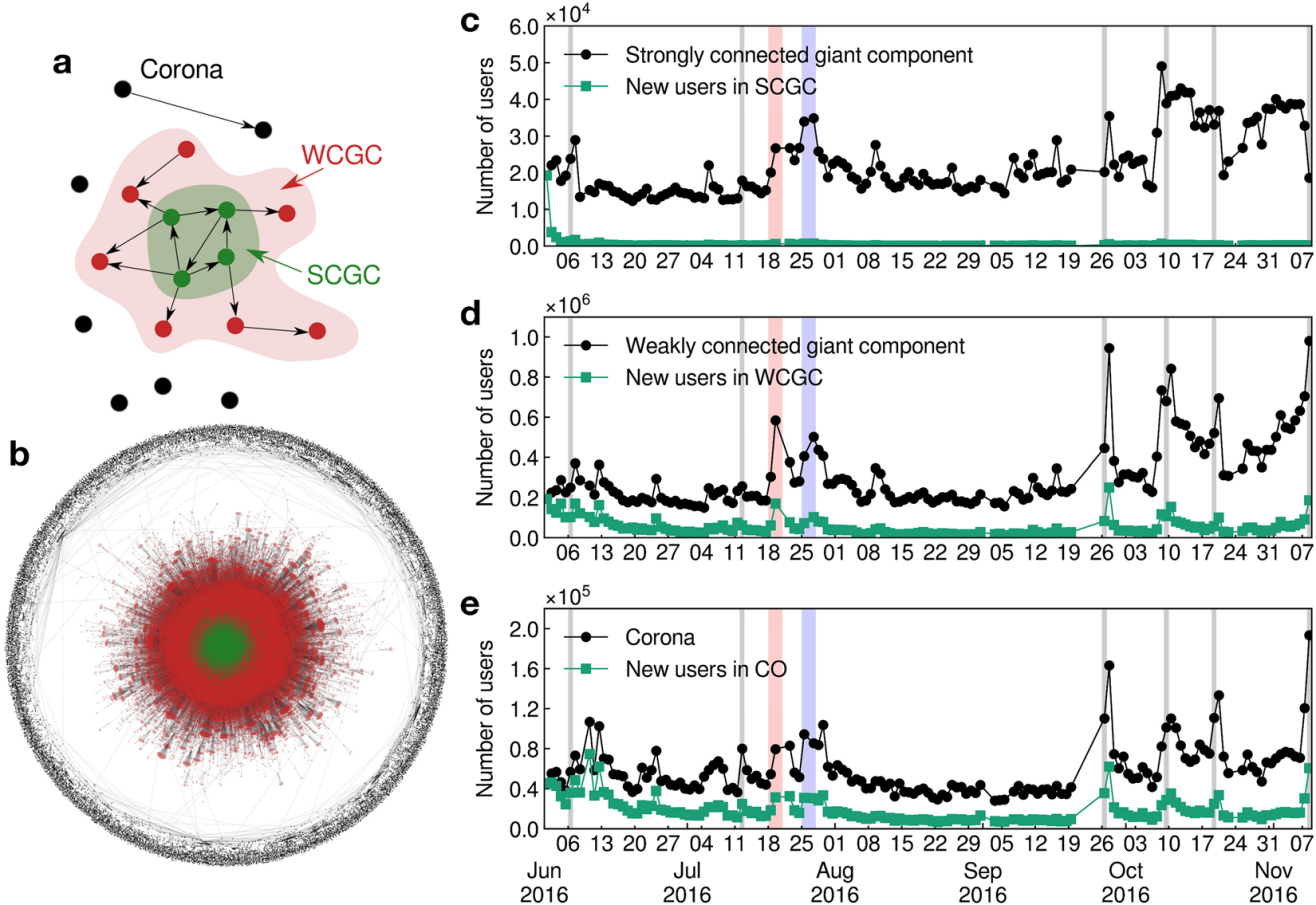
Temporal evolution of daily network components of Twitter election users. (**a**) Sketch representing the weakly (red) and strongly (green) connected giant components and the corona (black). (**b**) Influence daily network reconstructed from our Twitter dataset. The strongly connected giant component (green) is the largest maximal set of nodes where there exists a path in both directions between each pair of nodes. The weakly connected giant component (red) is the largest maximal set of nodes where there exists a path in at least one direction between each pair of nodes. The corona (black) is formed by the smaller components. (**c**) Total number of users in the daily strongly connected giant component (SCGC) versus time, (**d**) weakly connected giant component (WCGC) and (**e**) the corona, i.e. the rest of the components (displayed in black in (**a**) and (**b**)). The number of new users arriving in each compartment is shown in green. New users arrive principally in the weakly connected giant component or the corona. The shaded areas represents important events: the Associated Press announcing of Clinton winning the nomination (June 6), Bernie Sanders officially terminating his campaign and endorsing Clinton (July 12), the Republican (June 18–21) and Democratic (June 25–28) Conventions and the three presidential debates (September 26, October 9 and October 19).

We then build the daily social networks from user interactions following the methods developed in ref.^42^ (see Methods). A directed link between two users is defined whenever one user retweets, replies to, mentions or quotes another user. Using concepts borrowed from percolation theory^43,44^ we define different connected components to characterize the connectivity properties of the network of Twitter users: the strongly connected giant component (SCGC), weakly connected giant component (WCGC) and the corona (the rest of the network–composed of smaller subgraphs not connected to the giant components SCGC and WCGC). The SCGC is formed by the users that are part of interaction loops and are the most involved in discussions while WCGC is formed by users that do not necessarily have reciprocal interactions with other users (see Fig. 1a). A typical daily network is shown in Fig. 1b.

We monitor the evolution of the size of the SCGC, WCGC and the corona as shown in Fig. 1. The WCGC has an average daily size $\simeq$310,000 (standard deviation of 160,000 users) and is approximately 14 times larger than the SCGC with $\simeq$22,000 (standard deviation of 8,600) daily users (see Fig. 1c,d). The average daily number of users in the corona is approximately 58,000 with a standard deviation of 25,000 users (Fig. 1e). Fluctuations in the size of the three compartments are visible in the large spikes in activity occurring during important events that happened during the period of observation. For instance, on June 6, when Hillary Clinton secured enough delegates to be the nominee of the Democratic Party. Bernie Sanders (who was the second contender for the Democratic Nomination) officially terminated his campaign and endorsed Hillary Clinton on July 12. The Republican and Democratic Conventions were held between June 18–21 and June 25–28, respectively and the three presidential debates were held on September 26, October 9 and October 19. The fluctuations related to these events are more important in the WCGC and corona than in the SCGC. The number of new users–those appearing in our dataset for the first time–in each compartment is displayed in green in Fig. 1c–e. Most new users arrive and connect directly to the WCGC or populate the disconnected corona while relatively few users join directly the strongly connected component. This is expected as the users belonging to SCGC are those who are supposed to be the influencers in the campaigns, since for users in the SCGC, the information can arrive from any other member of the giant component, and, vice-versa, the information can flow from the member to any other user in the SCGC. Thus, it may take time for a new arrival to belong to the SCGC. After the first week of observation, the number of new users arriving directly to the SCGC per day stays stable below 1,000. Note also that, although Hillary Clinton and Donald Trump were officially nominated as presidential candidate of their respective party only during the conventions, they both secured enough pledged delegates to become the nominee before their party’s convention. Donald Trump secured enough delegates on May 26 while Hillary Clinton did it on June 6.

### Opinion of Twitter users

**Figure 2:**
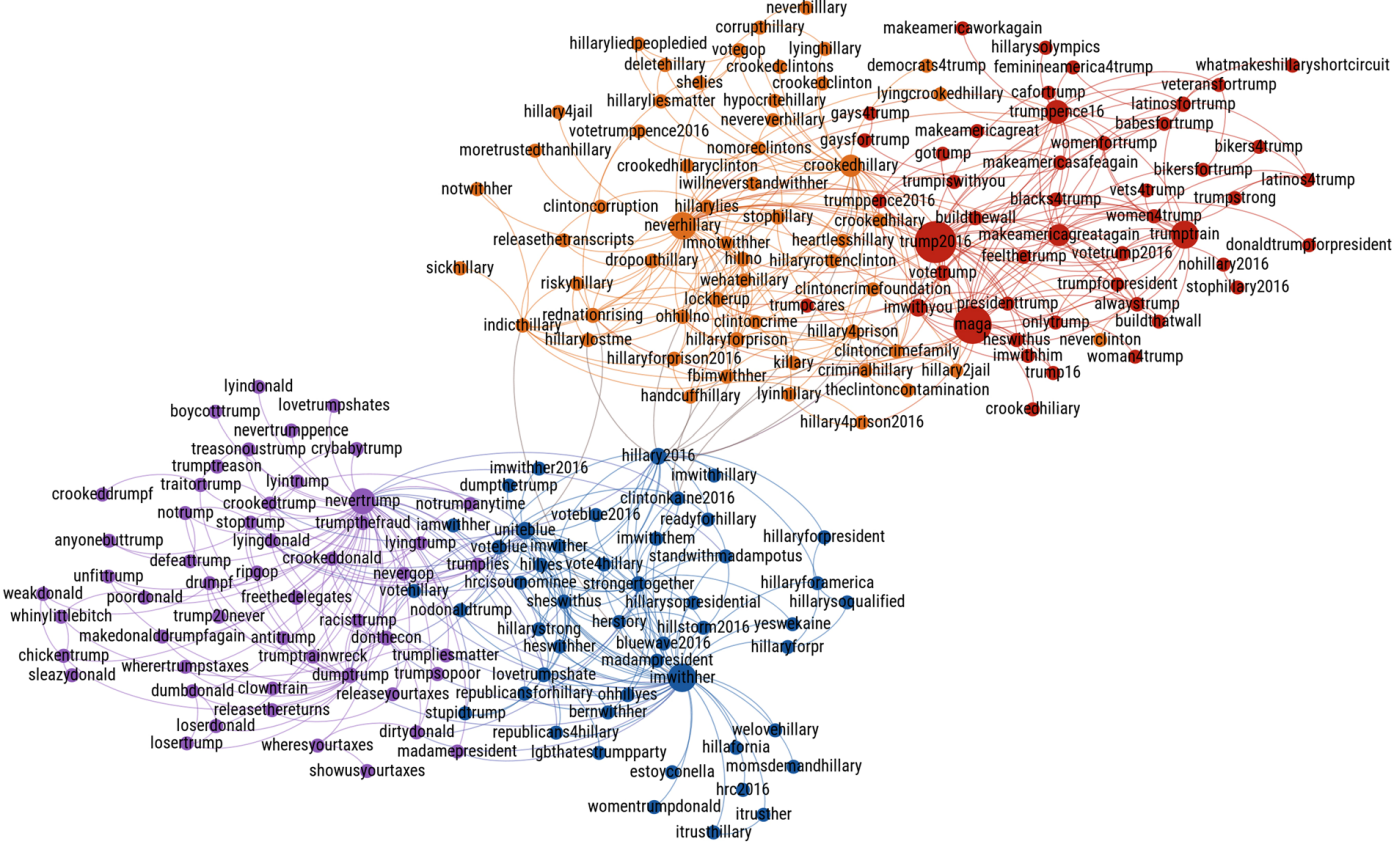
Hashtag classification via network of co-occurrence. Network of hashtags obtained by our algorithm from June 1st to September 1st. Nodes of the network represent hashtags and an edge is drawn between two hashtags when their co-occurrence in tweets is significant (see Methods). The size of the node is proportional to the total number of occurrence of the hashtag. Two main clusters are visible, corresponding to the Pro-Trump/Anti-Clinton and Pro-Clinton/Anti-Trump hashtags. Inside of these two clusters, the separation between Pro-Trump (red) and Anti-Clinton (orange), or Pro-Clinton (blue) and Anti-Trump (purple), is also visible. The coloring corresponds to clusters found by community detection^45,46^.

We use a set of hashtags expressing opinion to build a set of labeled tweets, which are used in turn to train a machine learning classifier (see Methods). Figure 2 displays the network of hashtags co-occurrence discovered with our algorithm from June 1st to September 1st. The network corresponding to the period from September 1st to November 8th displays similar characteristics and is shown in the Fig. S1 of the Supplementary Information. Hashtags are colored according to the four categories, pro-Trump (red), anti-Clinton (orange), pro-Clinton (blue) and anti-Trump (purple). Two main clusters, formed by the pro-Trump and anti-Clinton on the right and pro-Clinton and anti-Trump on the left, are visible, indicating a strong relation between the usage of hashtags in these two pairs of categories.

The existence of these two main clusters reveals the strong polarization of the opinion in our dataset and motivates our decision to reduce our classification of tweets to two categories: pro-Trump or anti-Clinton and pro-Clinton or anti-Trump, in the following designated as Trump supporters and Clinton supporters, respectively. The polarization can be quantified by the Newman modularity^47^ of the network partition, *Q*, which, in our case, measures the fraction of the co-occurrences between hashtags of the same group minus the expected value of the same quantity in a network with the same community divisions but random co-occurrences between the hashtags. For example, the modularity of the network in Fig. 2 partitioned in two groups (Clinton supporters versus Trump supporters) is *Q* = 0.48, while the modularity of the sub-graph consisting of only the hashtags of the Trump supporters partitioned in pro-Trump versus anti-Clinton is *Q* = 0.33 revealing a smaller degree of polarization.

The clear separation of hashtag usage in two main clusters corroborates previous studies showing that, in the case of political issues, Twitter users exchange primarily among individuals with similar ideological preferences as shown in Barbera *et al*.^48^. This result allows us to use the hashtags from the two clusters to create a labeled training set of tweets that is large enough (1 million tweets) so that the opinion of all the Twitter users (11 million) can be inferred with confidence such that it agrees with the NYT National Polling Average.

In principle, it may not be a given fact that any topic of interest could lead to the same separation in the hashtag network. We could imagine another topic of discussion in Twitter, let’s say, “Samsung Galaxy versus IPhone”, where users express their opinion about the two smartphones. In this case, one would be interested to see whether the co-occurrence hashtag network separates into two distinct clusters as in the case of Clinton/Trump. If this is the case, then it implies that the topic is polarized and our analytics can be applied confidently to obtain the number of users in favor of one smartphone or the other. However, it could be the case, that the hashtags do not separate into two groups. In this case, we would conclude that the topic is not polarized enough and therefore there are not well defined groups. Thus, the separation in the hashtag network is a necessary ingredient that allows to perform the supervised classification. The quality of the classification can be measured using classification scores during a cross-validation which permits to assess if the degree of polarization of the topic is sufficient.

Taking into account the entire observation period, we identify more hashtags in the pro-Trump (n = 60) than in the pro-Clinton (n = 52) categories and approximately the same number in the anti-Trump (n = 62) and anti-Clinton (n = 65) categories. The number of tweets using at least one of the classified hashtags account for 30% of all the tweets containing at least one hashtag. We find more tweets having hashtags exclusively in the Trump supporters category than exclusively in the Clinton supporters category (9.6 million for Trump versus 3.0 million for Clinton). These tweets also correspond to more users in the Trump camp than in the Clinton camp (538,720 for Trump versus 393,829 for Clinton). Although these figures might suggest a clear advantage for Trump in Twitter, one need to take into account the whole population of users in the dataset to correctly estimate the popularity of each candidate. This is what we show in the section “Measuring political support“, where the daily opinion of each users is determined after having classified our entire dataset of tweets.

### Generalization of the method to multi-partite elections and topics beyond elections

A two-classes classification scheme was the best approach in the case of the 2016 US Presidential elections since the elections where dominated by two candidates. However, in the case of multi-partite political systems like some European or Latin American countries, we can implement a multi-class classification scheme. This is done, for example, generalizing the binary classification used with Trump/Clinton to a multi-classification scheme involving three or more Parties.

The important ingredient for the application of the supervised learning technique to any kind of topic is the separation of the hashtag co-occurrence network in well-defined clusters identifying the main opinions toward the issue at hand. For instance, in the case of Trump/Clinton, the separation of the co-occurrence hashtag network into two clear camps allowed the application of the supervised method to the US Election with two main candidates. In the case of the 2017 French presidential election, we have collected tweets related to three main candidates (François Fillon, Marine Le Pen and Jean-Luc Mélanchon) to test the generality of our analytics to this kind of multi-partite political systems. The chosen candidates correspond to the three main confirmed candidates at the time of data acquisition (December 19th, 2016 to January 31st, 2017). This is the reason why we did not acquire data about Emmanuel Macron, who ultimately passed the first round of the elections along with Marine Le Pen. The results of the co-occurrence hashtag networks emanating from the tweets related to the French elections are shown in Fig. S6 in the Supplementary Information. We find that a separation of the co-occurrence network into three clear clusters is achieved for the hashtags employed by users expressing supports to three candidates to the French presidential election. Each group expresses predilection for each of the three French presidential candidates indicating that the opinion inference methods can be applied to this kind of situation as well.

Furthermore, we have also investigated whether the method can be implemented for public opinion outside an election setting. These “generality tests” are of importance to distinguish an ad-hoc research (anecdotal) versus a methodological contribution. For this purpose, we have collected tweets from a single topic, such as “climate change”, in search of a generalization of our algorithms. Figure S5 in the Supplementary Information shows the result of the hashtag co-occurrence network in this case. We find that this network naturally splits into two groups, one with hashtags supporting action toward climate change and the other with hashtags depicting climate change as a hoax. This result suggests that our machine learning and co-occurrence hashtag network method can be generalized to topics beyond the election setting. The minimal ingredients to apply our methods are the existence of a set of users interested in the topic and the appearance of separated hashtag clusters in the co-occurrence network. This separation was evident in all cases considered in this study: the US and French elections as well as climate change.

### Measuring political support

**Figure 3:**
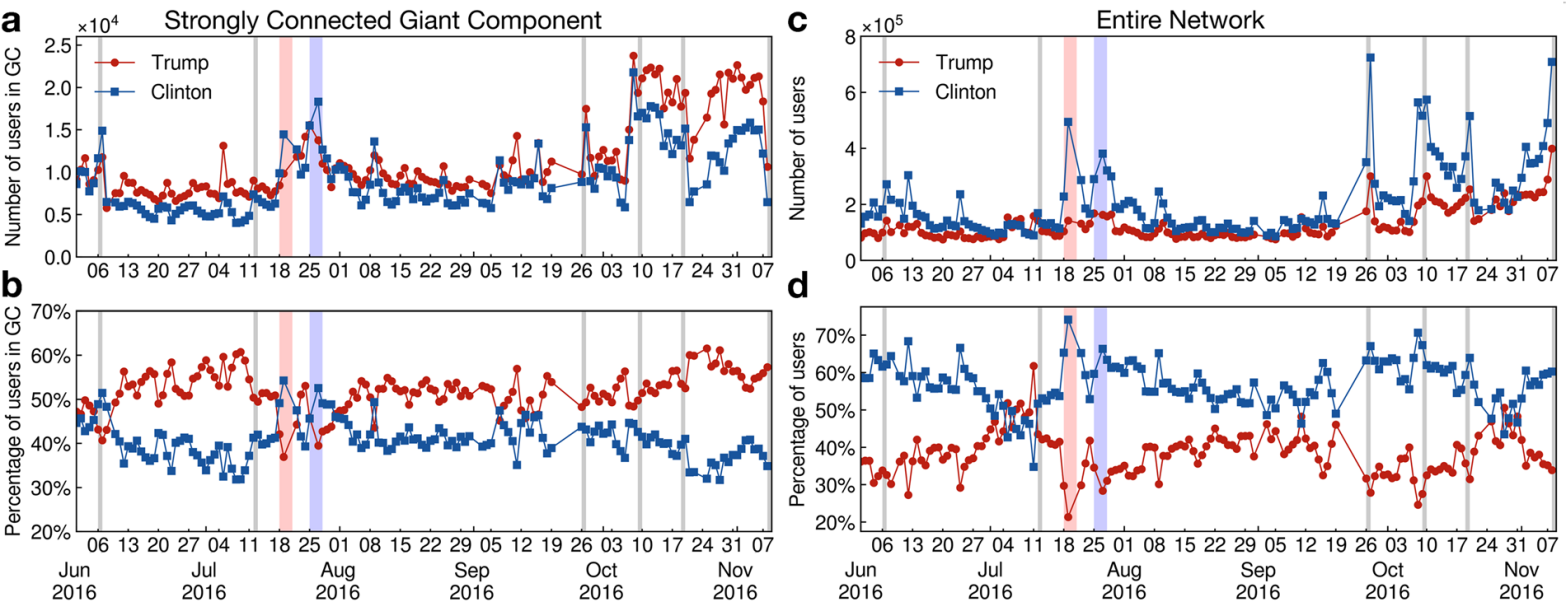
Supporters dynamics. (**a**) Absolute number and (**b**) percentage of supporters of Trump (red, Pro-Trump or Anti-Clinton) and Clinton (blue, Pro-Clinton or Anti-Trump) inside the strongly connected giant component as a function of time. Total number and (**c**) percentage of users (**d**) labeled as Trump (red) and as Clinton (blue) in our entire Twitter population as a function of time. The opinion in the strongly connected giant component is clearly in favor of Donald Trump and shifts slightly in favor of Hillary Clinton only occasionally, such as during the two Conventions. Taking into account all the users in our dataset, the popular opinion is generally strongly in favor of Hillary Clinton (**d**) in contrast with the strongly connected giant component (**b**). In (**b**) and (**d**) the data adds to 100% when considering the unclassified users ($\simeq$4.5%). The popularity of Donald Trump peaks before the conventions and before the election, however Hillary Clinton dominates Twitter opinion, in particular during important events, such as the conventions, the presidential debates and the election, coinciding with large positive fluctuations in the total number of users.

The absolute number of users expressing support for Clinton and Trump as well as relative percentage of supporters for each party’s candidate in the strongly connected component and in the entire population dataset is shown in Fig. 3. Results for the weakly connected component are similar to the whole population. The support of each users is assigned to the candidate for which the majority of its daily tweets are classified (see Methods). Approximately 4.5% of the users are unclassified every day, as they posts the same number of tweets supporting Trump and Clinton. We only consider tweets originating from official Twitter clients in order to discard tweets that might originate from bots and to limit the number of tweets posted from professional accounts (see methods). After this filtering, 92% of the total number of tweets remain.

We find important differences in the popularity of the candidates according to the giant components considered. The majority of users in the SCGC are clearly in favor of Donald Trump for the majority of the time of observation (Fig. 3b). However, the situation is reversed, with Clinton being more popular than Trump, when the entire Twitter dataset population is taken into account (Fig. 3d), revealing a difference in the network localization of the users belonging to the different political parties. The inversion of the opinion of the SCGC as compared with the result of the whole network allows us to reveal the behavior of Trump/Clinton voters in a way only possible through Twitter, since the network information is only available from Twitter but not from the National Polls. A difference in the dynamics of the supporters’ opinion is also uncovered: during important events, such as the conventions or the presidential debates, Hillary Clinton’s supporters show a much more important response than Donald Trump’s supporters (Fig. 3c) and even sometimes slightly dominate the SCGC (Fig. 3b). This difference in behavior is also manifested in the fact that spikes in favor of Donald Trump in the percentage of opinion (Fig. 3d), such as on October 28 when FBI Director, James B. Comey, sent a letter to the Congress saying that new emails, potentially linked to the closed investigation into whether Hillary Clinton had mishandled classified information, had be found, correspond rather to a lack of activity of Clinton’s supporters than to an increase in the engagement of Trump’s supporters (seen in Fig. 3c). We analyze these differences in behavior and their impact on the Twitter opinion trend in the section Analysis of Twitter supporters behavior.

### Comparison with national polls aggregates

We next compare the daily global opinion measured in our entire Twitter dataset with the opinion obtained from traditional polls. We use the opinion trend computed from the entire dataset (Fig. 3d) as it represents the average opinion of our entire user sample. We compare the Twitter opinion time series with the National Polling Average computed by the New York Times (NYT)^40^ which is a weighted average of all polls (n = 270) listed in the Huffington Post Pollster API (http://elections.huffingtonpost.com/pollster/api). Greater weights are given to polls conducted more recently and polls with a larger sample size. Three types of traditional polls are used: live telephone polls, online polls and interactive voice response polls. The sample size of each poll typically varies between several hundreds and tens of thousands respondents and therefore the aggregate of all polls considered by the NYT represents a sampling size in the hundred of thousand of respondents.

**Figure 4:**
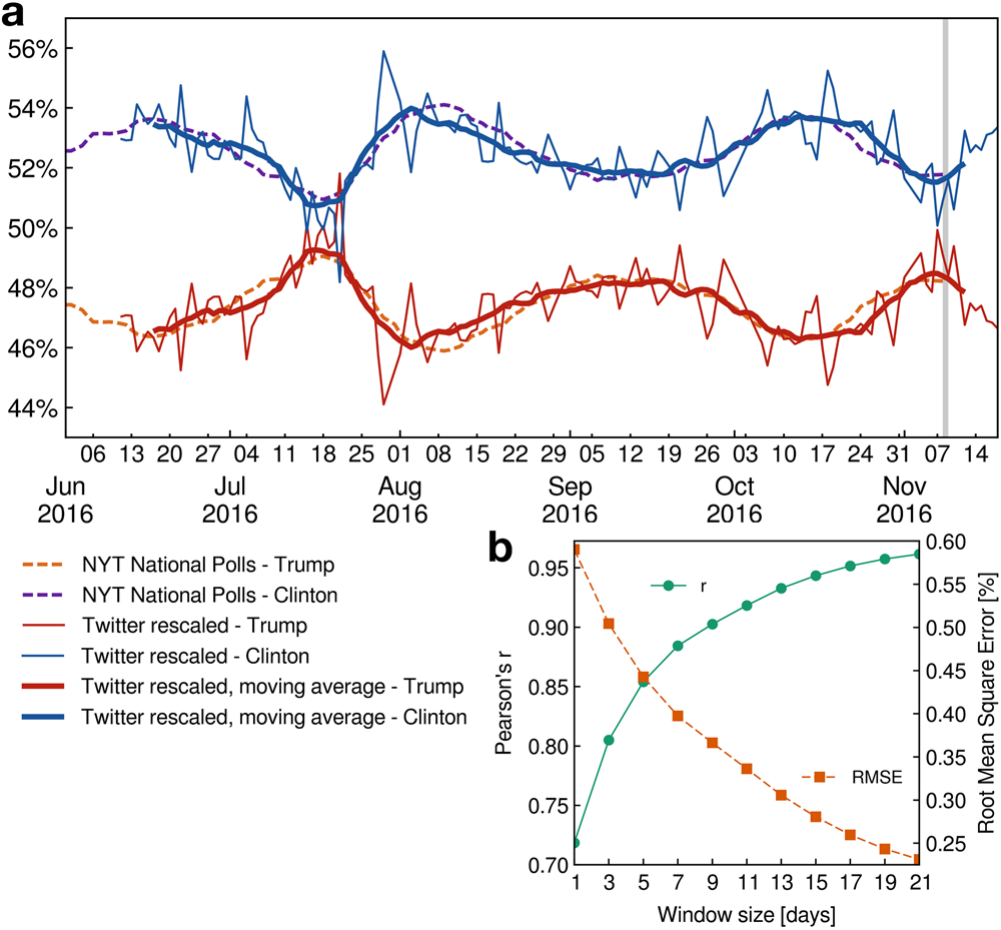
Validation of Twitter election trend and NYT aggregate national polls. (**a**) Least square fit of the percentage of Twitter supporters in favor of Donald Trump and Hillary Clinton with the results of the polls aggregated by the New York Times for the popular votes and normalized to the share of the two candidates. Twitter opinion time serie is in close agreement with the NYT National Polls. As Twitter provides an instantaneous measure of the opinion of its users, a time-shift of 10 days exists between the New York Times polls and the Twitter opinion. Pearson’s correlation coefficient (r) between the NYT and the 13 days moving averaged Twitter opinion has a remarkably high value $r\simeq 0.93$ with a root-mean-square error (RMSE) of $\simeq 0.31$%. (**b**) Pearson’*r* and RMSE of the fit as a function of the moving average window length. The Pearson coefficient, quickly increases and the root-mean-square error decreases as the window length increases and smooths out the daily fluctuations. The best fit is obtained for a window length of 21 days with RMSE $\simeq 0.23$% and $r\simeq 0.96$. RMSE is expressed in percentage point of the NYT polls.

We remove the shares of undecided and third party candidates from the NYT polling average and compare the resulting relative opinion trend with the ratio of Twitter users in favor of Donald Trump or Hillary Clinton. The comparison between our Twitter opinion, Fig. 3d, and the New York Times national polling average is shown in Fig. 4a. The global opinion obtained from our Twitter dataset is in excellent agreement with the NYT polling average. The scale of the oscillations visible in the support trends in Twitter and in the NYT polls are also in agreement beyond the small scale fluctuations which are visible in the Twitter opinion time series since it represents a largely fluctuating daily average. Furthermore, a time shift exists between the opinion in Twitter and the NYT polls in the sense that the Twitter data anticipates the NYT National Polls by several days. This shift reflects the fact that Twitter represents the fresh, instantaneous opinion of its users while traditional polls may represent a delayed response of the general population that takes more time to spread, as well as typical delays in performing and compiling traditional polls by pollsters.

In order to precisely evaluate the agreement between Twitter and NYT time series, we perform a least square fit of a linear function of the Twitter normalized ratio of supporters of each candidate to their NYT normalized popularity percentage (see Methods). Specifically, we apply the following transformation:

1 $${r^{\prime} }_{w}^{k}(i)\mapsto {A}^{k}\,\,{r}_{w}^{k}(i-{t}_{d})+{b}^{k},$$

where ${r}_{w}^{k}(i)$ is the ratio of Twitter users in favor of a candidate *k* (*k = C* for Clinton and *k = T* for Trump) at day *i* to which we applied a backward moving average with a window length *w*. The rescaling parameters *A*^*k*^ and *b*^*k*^ are the parameters that fit the NYT polls and *t*_*d*_ is a time delay between the Twitter opinion and the polls. The moving window average of *w* days converts fluctuating daily data into a smooth trend that can be compared with the NYT smooth time series aggregated over many polls performed over several days. We use a backward window average to ensure that no data from the future is used. Note that a backward moving average induces an artificial backward time shift of (*w* − 1)/2 (see Methods) so that the full time shift between the Twitter time-series and the NYT polls that we report below is given by *T*_*d*_ = *t*_*d*_ + (*w* − 1)/2.

Figure 4a shows the fit using a window averaging of the Twitter data of 13 days. The constant parameters that provide the best fit in this case are *A*^*C*^ = *A*^*T*^ = 0.185, *b*^*C*^ = 1 − *b*^*T*^ − *A*^*T*^ = 0.415, *T*_*d*_ = 10 days. The remarkable agreement of the fit is characterized by a Pearson product-moment correlation coefficient *r* = 0.93 and a root-mean-square error of RMSE = 0.31%, expressed in percentage points of the NYT polls. Using longer window average length increases the quality of the fit as shown in Fig. 4b displaying the root-mean-square error expressed in percentage points of the NYT polls and the Pearson correlation coefficient of the fit as a function of the moving average window length *w*.

It is important to note that Twitter data cannot predict the exact percentage of supporters to each candidate in the general population due to the uncertainty about the number of voters that do not express their opinion on Twitter and about the number of users that are undecided and are not classified by our machine learning. However, it is more important to capture the trend of both candidates’ popularity in respect to each other, which is obtained from Twitter with precision. Furthermore, an important parameter is *T*_*d*_, the time delay between the anticipated opinion trend in Twitter and the delayed response captured by the NYT population at large. We find that this delay time is independent from the actual value of the popularity of each candidate and from the length of the window average.

Next, to assess the performance of our algorithm in a more realistic context, when the only accessible data is retrospective, we train it only during the three first months of our dataset and evaluate the agreement until election day, i.e. we train our classifier using tweets labeled with hashtags found with the hashtag co-occurrence network (Fig. 2), compute the daily ratio of users in favor of each candidate and find the parameters *A*, *b* and *T*_*d*_ that best fit the NYT polls, using only the portion of our data ranging from June 1st until September 1st. We then classify the rest of the tweets with the pre-trained classifier and keep the same fitting parameters to compare our Twitter trend with the NYT polls until election day on November 8th. We compare this results with a straightforward extrapolation in time of the NYT polls from a linear regression on the last three weeks of the polls, as in Beauchamp^35^, a constant extrapolation using the mean value of the polls during the training period and a more advanced model using a Autoregressive integrated moving average^49^ (ARIMA) to forecast the polls. We choose the order, (*p*, *d*, *q*), of the ARIMA (*p*, *d*, *q*) model by maximizing the loglikelihood of the fit with the three first months of the NYT polling average and select the best parameters by minimizing the Akaike information criterion^50^. In a ARIMA (*p*, *d*, *q*) model, *p* is the order of the autoregressive model, *d* is the degree of differencing and *q* is the order of the moving-average model. We find that the model best representing the polls is a ARIMA(4, 1, 3). We then fit this model to the polls from the start of the observation period until day *D* and use it to forecast the polls at day *D* + 7 until election day.

**Figure 5:**
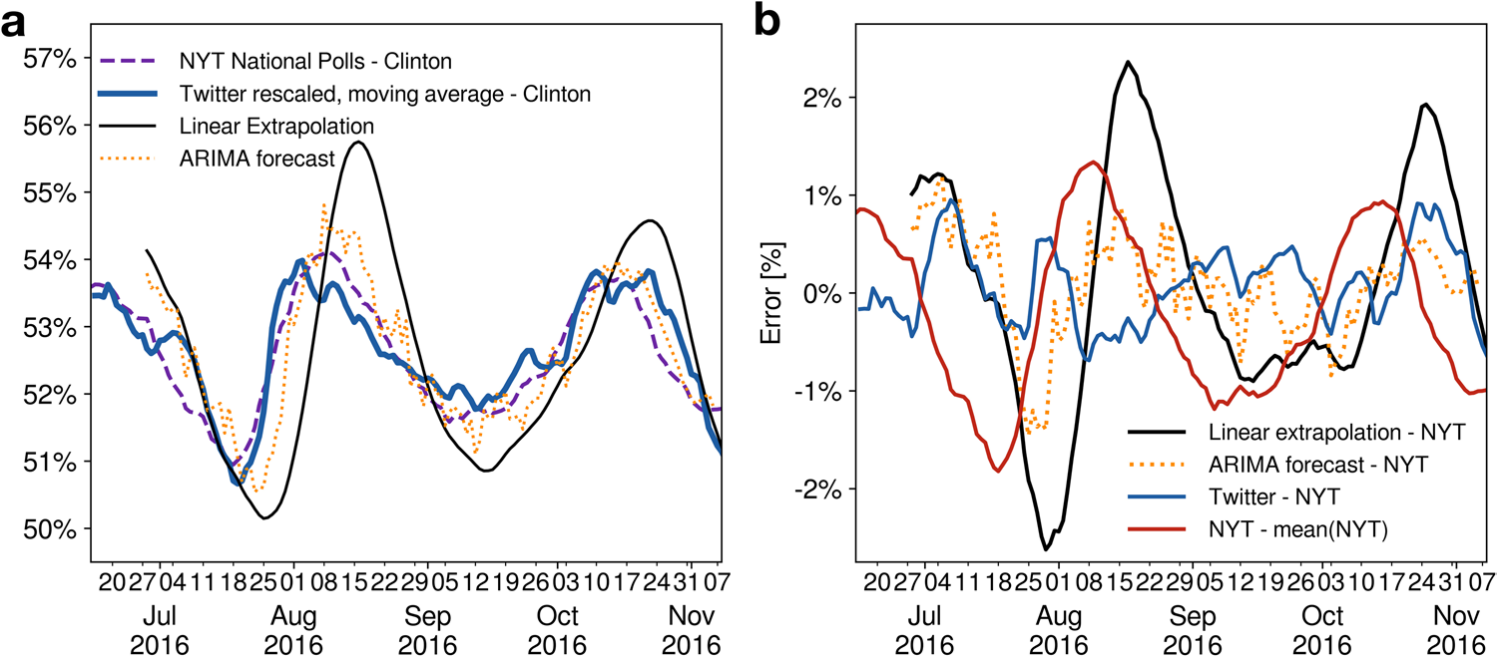
Twitter estimation of the polls 7 days in advance versus linear extrapolation of the polls. (**a**) Twitter estimation of the NYT polls 7 days in advance (blue line), 7 days linear extrapolation of the NYT polls (black line), 7 days forecast using a ARIMA model (dotted orange line) and Hillary Clinton NYT National Polling Average score, normalized to the share of Donald Trump and Hillary Clinton, (dashed purple line). Our model is trained using only data from June 1st to September 1st. (**b**) Estimation error in percentage points of the NYT polls. The Twitter estimation error (blue) has a root-mean-square value of RMSE = 0.40% (correlation coefficient *r* = 0.89). The 7 days linear extrapolation of the polls (black) has a RMSE = 1.19% (*r* = 0.64), The 7 days ARIMA forecast (orange) has a RMSE = 0.33% (*r* = 0.91) and the baseline error, computed as the difference between the NYT Polling Average and the its mean value (red), achieves a RMSE = 0.83%.

Training our model only on the first three months, the time delay giving the best fit is *T*_*d*_ = 11 days. We use our Twitter trend smoothed with a 9 days backward moving average to estimate the polls 7 days into the future (see Methods). We find that our Twitter opinion matches the NYT polls with better accuracy, i.e. a smaller root-mean-square error (RMSE), than a straightforward linear extrapolation and a simple constant extrapolation using the mean value of the polls (see Fig. 5). Estimating the polls 7 days in advance, we find that the Twitter opinion reduces the RMSE by 52% compared to the constant extrapolation and by 66% compared to the linear extrapolation. The estimation error of the different methods are displayed in Fig. 5b. Even more importantly, our Twitter opinion is particularly better than the linear extrapolation to detect rapid changes in the polls as seen in Fig. 5a which shows the estimation from Twitter along with the result of the linear extrapolation. Although the linear extrapolation unsurprisingly predict relatively well the polls when they undergo small variations, when the polls experience a trend reversal, the Twitter opinion accurately estimate it while the linear extrapolation misses it. The more advanced ARIMA forecast based on the polls performs better than the comparison based on our Twitter opinion time series with a RMSE = 0.33% (compared to RMSE = 0.40%). However, an important distinction between forecasting using methods based on the polls themselves (linear extrapolation or ARIMA regressions) and the comparison we made using Twitter is that, in the case of the Twitter opinion time series, the polls are only used to find the scaling parameters during the first portion of the dataset and then it still accurately matches the traditional polls during the remaining part of the dataset for more than two months. Other forecasting methods continuously use the polls to predict their values 7 days in advance. The ability of our method to estimate the polls with an accuracy close to a ARIMA forecast during more than two months without reusing the polls to retrain our model, serves as robust validation of the lag observed between Twitter opinion and the traditional polls.

### Analysis of Twitter supporter behavior

We showed that the variations of opinion measured in Twitter are in very good agreement with the variations of relative opinion in the NYT National Polling average, and, as for the NYT polls, the majority in Twitter is in favor of Hillary Clinton. Here, we show that Twitter can also be used to analyze the behavior of the supporters in order to understand the dynamics of its opinion trend, something not possible with traditional polls.

**Figure 6:**
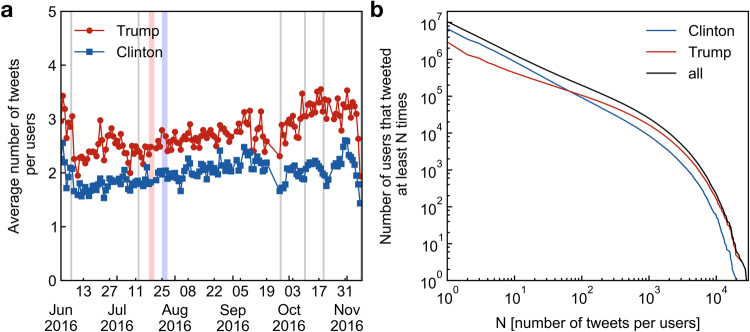
Activity of Twitter supporters. (**a**) Daily average number of tweets per user of each camp. Trump supporters have a higher average activity (shown in red), tweeting on average 3.9 times per day while Clinton supporters tweet on average 2.6 times (show in blue). (**b**) Distribution of user activity. Activity of all users (black), Clinton supporters (blue) and Trump supporters (red). The distributions are characterized by a power-law shape with a soft cut-off around 1000 tweets per users. The distribution for Clinton supporters has a steeper power-law indicating a generally smaller activity than Trump supporters.

Firstly, in addition to measuring the daily opinion, we measure the opinion of the entire population of Twitter users whose tweets we collected over the period going from June 1st until November 8th, something not possible for the majority of traditional polls. That is, using all the tweets in our dataset posted by a users over the entire observation period, we classify each users according to camp in which the majority of his/her tweets is classified. This calculation contrast to the one employed by polls, which can track only a sample population at a given time. In this cumulative count, each user is only counted once, while in the daily count, a user is counted every day she/he expresses her/his opinion in Twitter. Considering this cumulative count, we find that a large majority of users, 64%, is in favor of Hillary Clinton while 28% are in favor of Donald Trump and 8% are unclassified as they have the same number of tweets in each camp. The cumulative count contrasts with the daily count. The average of the daily opinion over the same period (Fig. 3d) amount to 55% for Hillary Clinton versus 40% for Donald Trump (5% unclassified). Such a large difference between the daily and the cumulative ratios of supporters indicates a difference in average activity of the supporters of each camp. Indeed, looking at the activity of users in both camps we see that Trump supporters are, in average, much more active. Figure 6a shows the daily average of tweets per users in each camp. Clinton supporters tweet in average 2.6 times per day while Trump supporters tweet in average 3.9 times per day and their activity increases to almost 4 tweets per day during the period of the presidential debates.

The cumulative distribution of the number of times a user tweets for each camp (Fig. 6b) reveals a clear difference in the activity profiles of supporters of each camps. While both distributions follow a power-law form with a soft cut-off starting around 1000 tweets per users, the distribution of Trump users shows a less steeper slope than the one for Clinton supporters revealing that Trump supporters are generally characterized by a larger activity and that a small number of Trump supporters have a extremely high activity. Figure 6b shows that by considering only users for which we collected at least 67 tweets during the entire observation period, the advantage tilts in favor of Donald Trump. This discrepancy in activity between supporters is also apparent in the structure of the social network where the strongly connected giant component, which show less size fluctuations than the other components and is mainly comprised of recurring users, is dominated by Trump supporters (Fig. 3b) in clear opposition to the entire dataset (Fig. 3d).

A second observation available from our Twitter analysis that is not available to traditional pollsters is the difference in the dynamics of supporters of each camp. Figure 3c shows that the daily number of Trump supporters fluctuates less than the daily number of Clinton supporters. We find that ${\sigma }_{{n}_{C}}/{\sigma }_{{n}_{T}}\simeq 2.1$ where ${\sigma }_{{n}_{k}}$ is the standard deviation of the daily number of users in favor of user *k*. Trump supporters show a more constant support while Clinton supporters show their support mainly when important events occur, leading to larger fluctuations in their daily absolute number. To understand the impact of these different behaviors on the value of the ratio of users in favor of each candidate, we evaluate Spearman’s rank correlation coefficients between the daily value of the absolute number of users in favor of a candidate (Fig. 3c) and the ratio of user in favor of the same candidate (Fig. 3d). We find a value of ${\rho }_{C}\simeq 0.72$ for Clinton supporters and ${\rho }_{T}\simeq -\,0.28$. This results in the important fact that the relative variations of the daily opinion that we measure on Twitter (Fig. 3d), which agrees with the NYT polling average, are mainly explained by the variation of the support of Clinton supporters and almost not by the variation of the support of Trump users. Moreover, the negative value of *ρ*_*T*_ indicates that a positive fluctuation in the number of Trump supporters is generally correlated with even larger increase in the number of Clinton supporters. This analysis shows how opinion trends measured in Twitter can be understood as the result of the dynamics of the different supporters camp. The opinion trend mainly reflects the daily fluctuations of the Clinton supporters coming in and out of the sampled population.

### Benchmark with other Twitter-based metrics

Here we compare the performance of our method with the approaches used previously. Similarly to the approach we used to fit our Twitter opinion time series to the normalized NYT national polling average scores of the two main candidates, we build metrics ${M}_{l}^{k}$ for each approach *l*, where *k* ∈ (*C*, *T*) represent the candidate (*C* for Clinton and *T* for Trump), such that ${M}_{l}^{C}(i)+{M}_{l}^{T}(i)=1$ for each day *i*. Since the metrics are complementary in respect to each candidate, we only need to compare the metric for one candidate with the normalized poll scores of the same candidate.

The first approach consists in simply counting the number of users mentioning each candidate per day. This approach used by many authors (e.g.^10,11,19,21,25,29^) is generally thought to measure attention toward a candidate rather than opinion^20,29^. O’Connor *et al*.^10^ reported a correlation of *r* = 0.79 between the number of tweets per day (using a 15 days window average) mentioning Barack Obama and his score in the polls during the 2008 US Presidential elections. However, the authors found that the McCain 15-day mention volume also correlated to higher Obama ratings. Jungherr *et al*.^29^ reported correlations between the number of mentions per day of different parties and their polls scores during the 2013 German federal elections. The largest correlation being *r* = 0.279 for the party “Alternative für Deutschland” (with a time lag of 1 day between the polls and the Twitter metric). We compare the time series given by

2 $${M}_{{\rm{mentions}}}^{C}(i)=\frac{{N}_{u}^{C}(i)}{{N}_{u}^{C}(i)+{N}_{u}^{T}(i)},$$

where ${N}_{u}^{k}(i)$ is the number of users mentioning candidate *k* during day *i*, with the normalized poll score of Hillary Clinton. We use the keywords *donald*, *trump*, *donaldtrump* and *realdonaldtrump* to count mentions of Donald Trump and *hillary*, *clinton* and *hillaryclinton* for mentions of Hillary Clinton.

The second metrics we use consists of adding a sentiment analysis to the mention counts. This approach has also been wildly used (e.g.^10,12,13,17,25,29,34^) by inferring the sentiment of a tweets using lexicons^10,17,25^ or supervised-learning^13,34^. O’Connor *et al*.^10^ reported a smaller correlation for Obama taking into account sentiment (*r* = 0.44) compared to just counting mentions and a correlation of *r* = 0.731 for the sentiment of the keyword *jobs* with the time series of the consumer confidence (using a 15 days window average). For comparing with the polls, we define the metrics

3 $${M}_{\mathrm{mentions}-\mathrm{emotion}}^{C}(i)=\frac{{N}_{u}^{{\rm{C}},\mathrm{pos}}(i)+{N}_{u}^{{\rm{T}},\mathrm{neg}}(i)}{{N}_{u}^{{\rm{C}},\mathrm{pos}}(i)+{N}_{u}^{{\rm{C}},\mathrm{neg}}(i)+{N}_{u}^{{\rm{T}},\mathrm{neg}}(i)+{N}_{u}^{{\rm{T}},\mathrm{pos}}(i)},$$

where ${N}_{u}^{k,e}(i)$ is the number of users that mentioned candidate *k* in a tweet with sentiment *e*. To infer the sentiment *e* ∈ (*pos*, *neg*) of a tweet we trained a classifier on a training set comprising tweets from our datasets with positive and negative emoticons and emojis. This is similar to the method used in ref.^13^. We use supervised learning instead of a lexicon based approach due to the poor performance of such approach on the informal text of tweets^33^. We use a tweet-level classification instead of an approach allowing to infer directly the aggregated tweet sentiment values^34,37,39^ in order to be able to compute the user ratio in each camp, required to compare with our results.

The third metrics we consider is derived from the number of hashtags referring to the candidates. We define the metric as

4 $${M}_{{\rm{hashtags}}}^{C}(i)=\frac{{N}_{u}^{\mathrm{pro} \mbox{-} {\rm{C}}}(i)+{N}_{u}^{\mathrm{anti} \mbox{-} {\rm{T}}}(i)}{{N}_{u}^{\mathrm{pro} \mbox{-} {\rm{C}}}(i)+{N}_{u}^{\mathrm{anti} \mbox{-} {\rm{C}}}(i)+{N}_{u}^{\mathrm{pro} \mbox{-} {\rm{T}}}(i)+{N}_{u}^{\mathrm{anti} \mbox{-} {\rm{T}}}(i)},$$

where ${N}_{u}^{\mathrm{pro} \mbox{-} k}(i)$, respectively ${N}_{u}^{\mathrm{anti} \mbox{-} k}(i)$, is the number of users using at least one hashtag in favor of, respectively in opposition to, candidate *k* during day *i*. To represent each category, we use hashtags chosen among the top used hashtags: *#MAGA* for pro-Trump, *#ImWithHer* for pro-Clinton, *#NeverTrump* for anti-Trump and *#NeverHillary* for anti-Clinton. This set is the same set that we used as a seed in the co-occurrence hashtag network for our hashtag discovery algorithm (see Methods). By counting hashtags and hashtags with positive or negative values associated with a party, Jungherr *et al*.^29^ reported a maximum absolute correlation of *r* = −0.564 for the party “Die Grüne” during the German 2013 federal elections (with a negative lag of 1 day between the polls and the Twitter time series).

For the fourth metrics, we train our classifier following the same procedure than for our Twitter opinion (see Methods) but using a training set built using only four hashtags selected among the top occurring hashtags (*#MAGA* for pro-Trump, *#ImWithHer* for pro-Clinton, *#NeverTrump* for anti-Trump and *#NeverHillary* for anti-Clinton). This metrics is representative of recent, state-of-the-art methods, used, for example, to mine to opinion about the “Brexit” in Twitter^51^. In ref.^51^, Amador *et al*. find a correlation of *r* = 0.852 between the tweet volume corresponding to tweets supporting the “Leave the EU” camp and a moving average of polls conducted to adults/likely voters using a classifier trained on tweets containing hashtags expressing supports for the “Leave” or “Remain” camps. We also show the results in term of classification scores (see Table 1) and in term of agreement with traditional polls when we fit this metrics to the NYT polling average (see Fig. S4 of the Supplementary Information).

**Figure 7:**
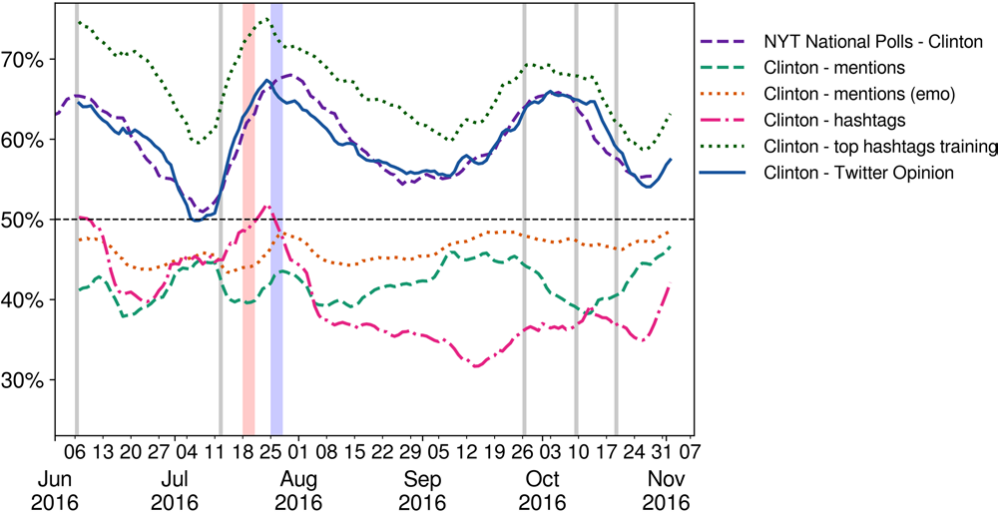
Comparison of the fit between different Twitter-based metrics and the NYT national polling average time series for Hillary Clinton. We show the normalized NYT poll scores of Hillary Clinton (dashed purple) fitted to our Twitter opinion metrics. The 13 days moving average of Hillary Clinton’s score computed using mentions (eq. 2, dashed green line), mentions with sentiment (eq. 3, dotted orange line), hashtags (eq. 4, dash-dotted pink line), a classifier trained using only the tweets containing the top hashtags in each camp (dotted green line) and our Twitter opinion (continuous blue line). All the metrics, except for our opinion metrics and the one obtained from the classifier trained using only top hashtags, are mainly below the 50% line, and therefore disagree with the NYT national polling average.

Figure 7 shows the results of the four time series obtained with these metrics, along with the result of our Twitter opinion time series, using a moving window average of 13 days in each case. The quality of the agreement that we find using our analytics between the national polls and Twitter trends is superior to previous approaches. The first three methods benchmarked agree poorly with the National Polls, as can be seen in Fig. 7. This evidence shows that the opinion in Twitter is different than the attention, which is what was measured in previous studies^10,13,17,29^. The fourth method, using only the top hashtags for building the training set, captures the NYT opinion trend, however using our method, with the full hashtag set, improves classification scores (see Table 1), increases the correlation with the polls and reduces the root-mean-square-error of the fit with the polls (see Fig. S4 of the Supplementary Information). These results confirm the importance of correctly measuring opinion in Twitter by assessing the supports of each user, something we achieve using a new method utilizing supervised machine learning with an in-domain training set of 1 million tweets built from the hashtags carrying an opinion.

## Discussion

Using Twitter as a sensor for public opinion has attracted enormous attention because there is a general sense that digital data may, at some point by the virtue of reaching much larger populations, outdate more traditional approaches to polls, which is of interest to social science and beyond. Indeed, there has been many contributions in journals^11,14,20,21,23,24,34,35,37,38,52,53^ and, in social computing conferences^10,12,13,15–19,22,25–27,54^ dealing with public opinion and political processes in Twitter.

The new method we present uses a combination of statistical physics of complex networks, natural language processing and machine learning to uncover the opinion of Twitter users and to analyze their behavior giving unique insights into the reason for the observed opinion variations. We find a remarkably high agreement between our measured Twitter opinion trend and the New York Times polling national average. The opinion trend in Twitter is instantaneous and anticipates the NYT aggregated surveys by 10 days. The agreement still holds when we train our data only on the first part of our dataset and evaluate it on the entire period of observation.

While the opinion we measure in Twitter supports the result of the US national popular vote, where Hillary Clinton won by a few percentage points, It is unlikely that Twitter alone could be used to predict the outcome of the election, taking into account the Electoral College system, due to its imbalanced demographic representation of the electorate^55^. However, as we showed, our method allows to capture national opinion trends from Twitter.

Our findings demonstrate that measuring the attention toward candidates does not allow to differentiate the political support toward each candidate. Indeed, a comparison with previously proposed methods^10,13,17,29^ based on the ratio of users mentioning each candidate shows a worse agreement with the NYT polling average than our Twitter opinion, even when sentiment analysis is used to classify tweets as positive or negative or when hashtags are used to classify users (see Fig. 7).

Furthermore, we showed the necessity of understanding the impact of the difference in activity of each supporters group on the final opinion trend to correctly interpret it. Our results reveal a difference in the behavior of Twitter users supporting Donald Trump and users supporting Hillary Clinton. Peaks in the opinion in favor of Clinton are highly correlated with large positive fluctuations in the daily number of Clinton supporters and coincide with important events such as the conventions or the presidential debates. On the other hand, peaks in favor of Trump correspond to a lack of mobilization of Clinton supporters. Although Clinton supporters are the majority in Twitter, Trump supporters are generally more active and more constant in their support, while Clinton supporters are less active and show their support only occasionally. This dichotomy is also visible in the user network dynamics. The strongly connected giant component (SCGC), dominated by Trump supporters, shows only small size fluctuations and comprises almost only recurring users, as opposed to the rest of the network, dominated by Clinton supporters, which shows large fluctuations and is where new users arrive. These findings confirm previous studies suggesting that right-wing leaning Twitter users exhibit greater levels of activity and more tightly interconnected social structure^18,56^. We push these observations further by showing how these effects influence opinion trend in Twitter. Indeed, our analysis shows that Twitter opinion is mainly measuring the reaction of Clinton supporters.

Our results validate the use of our Twitter analytics machinery as a mean of assessing opinions about political elections and show the necessity of accompanying the Twitter opinion with an analysis of user activity in order to correctly interpret its variations. Our analysis comes at a fraction of the cost of traditional NYT polling methods employed by aggregating the whole of the US$ 18 billion-revenue market research and public opinion polling industry (NAICS 54191). In contrast to traditional unaggregated polling campaigns which are unscalable, typically ranging at most in the few thousand respondents, our technique has the advantage of being highly scalable as they are only limited by the size of the underlying social networks. Moreover, traditional polling suffers from a declining rate of respondents being only 9% according to current estimates (2012) down from 36% in 1997^55^, while social media is gaining billions of users worldwide. Although the demographics representation of Twitter is biased^57^ and Twitter’s API introduces a supplementary unknown bias in our sample^33,58^, Twitter allows to study the behavior of its users and to understand the link between their activity and the variations in opinion trend, something not accessible to traditional polls.

Provided a large usage of opinion-hashtags and a polarization of opinion resulting in well separated hashtag clusters, our approach can be extended to understand other kind of trend from social media ranging from the opinion of users regarding products and brands, to other political movements, thus, unlocking the power of Twitter to understand trends in the society at large.

## Methods

### Data collection and social network reconstruction

We continuously collected tweets using the Twitter Search API from June 1st, 2016 to November 8th, 2016. We gather a total of 171 million tweets in the English language, mentioning the two top candidates from the Republican Party (Donald J. Trump) and Democratic Party (Hillary Clinton) by using two different queries with the following keywords: *trump OR realdonaldtrump OR donaldtrump* and *hillary OR clinton OR hillaryclinton*. During this period of 161 days, 15 days are missing due to connection errors.

To assess the importance of the possible noise in the data induced by the “trump” and “hillary” keywords, we filtered our dataset to keep only tweets with either one of the following keywords: ‘realdonaldtrump’, ‘hillaryclinton’, ‘donaldtrump’ or at least one of the following pairs of keywords ‘trump’ and ‘donald’ or ‘hillary’ and ‘clinton’. Although this keyword filtering reduces the dataset from 158 millions tweets to 58 millions tweets (considering only tweets from official clients), our results are not significantly changed, as shown in Fig. S2 in the Supplementary Information, and our conclusions still hold.

For every day in our dataset, we construct the social network *G*(*V*, *E*) where *V* is the set of vertices representing users and *E* is the set of edges representing interactions between the users. In this network, edges are directed and represent influence. When a user *v*_*i*_ ∈ *V*, retweets, replies to, mentions or quotes any other user *v*_*j*_ ∈ *V*, a directed edge is drawn from *v*_*j*_ to *v*_*i*_. We remove Donald Trump (*@realdonaldtrump*) and Hillary Clinton (*@hillaryclinton*) from the network, as we are interested by the opinion and dynamics of the rest of the network. We divide the network in three compartments: the strongly connected giant component (SCGC), the weakly connected giant component (WCGC) and the corona (Fig. 1). The SCGC is defined as the largest maximal set of nodes where there exists a path in both directions between each pair of nodes. The SCGC is formed by the central, most densely connected region of the network where the influencers are located, and where the interactions between users are numerous. The WCGC is the largest maximal set of nodes where there exists a path in at least one direction between each pair of nodes. The corona is formed by the smaller components of remaining users and the users that were only connected to Hillary Clinton or Donald Trump official accounts, which were removed for consistency. Users that do not interact with anyone else are not counted in the network, although we take them into account when computing the opinion of the entire dataset (see Fig. 3c,d).

### Filtering of automated tweets

To remove tweets that may originate from bots, we extracted the name of the Twitter client used to post each tweet from their *source* field and kept only tweets originating from an official twitter client. When a programmatic access to Twitter is gained through its API to send tweets, the value of the *source* field of automated tweets corresponds to the name, which must be unique, given to the “App” during the creation of access tokens^59,60^. Table S1 of the Supplementary Information shows the clients we considered as official and the corresponding number of tweets originating from each client. The number of tweets originating from official clients represent 92% of the total number of tweets. This simple method is well suited for our dataset as it discriminates at the tweet level, can be applied to historical data and scales very easily to large datasets contrary to more sophisticated methods^61^. It may however reject some tweets written from real users as real users may use non-official clients to manage their tweets and automate their activity to a certain degree. This is the case, for example, of some accounts belonging to professional users such as journalists, news outlets or brands. Examples of client names that we remove include: SocialFlow, Hootsuite, DropOuthillary1 and uZxbtLFGbRXiIBN5vUPT6gkuiTc3jfv. SocialFlow and Hootsuite correspond to third party applications that allow to automate some part of a user activity while the other clients do not correspond the any known third party applications and seem to be malicious.

In order to have a better understanding of the relation between the method we use to remove automated tweets and results of the state-of-the-art bot detection methods, we perform a comparison on a small test set using Botometer^61^. Botometer is machine learning framework using more than a thousand features extracted from public data and meta-data about users (friends, tweet content and sentiment, network patterns, and activity time series) to detect Twitter bots^61^. Varol *et al*. report a classification performance of 0.95 AUC (Area Under the receiver operating characteristic Curve). Note that a fair comparison with Botometer is not possible since Botometer is not well suited for historical data as it requires several tweets per users (up to 200) and results of a Twitter search of tweets (up to 100) mentioning each users. Moreover, Botometer classifies at the user level whereas our method classifies at the tweet level. The comparison we show in the following serves only as an indication of the relation between the two methods. We extract from our dataset 200 random user ids from the set of users that uses official clients and 200 random user ids from the set of users that have used, at least once, an unofficial client. We then use the free Botometer API (https://github.com/IUNetSci/botometer-python) to calculate the Botometer bot scores of each users in our test set. In order to compare Botometer’s results with our method, we collected the 200 latest tweets of each user in our test set (using Twitter API) and computed the ratio of tweets sent from an unofficial client for each user. Among the 400 users in our test set, 345 were not deleted from Twitter. Among the resulting 345 users, 60 have a Botometer english bot score above 0.5, a threshold recommended to distinguish advanced and simple bots from real users^61^, and 120 have a ratio of tweets sent from unofficial clients larger than 0.5. Using the results of Botometer as ground truth, we find that considering a ratio of unofficial clients larger than 0.5 to classify a user as bot results in an accuracy of 0.74, a recall of 0.77 and a precision of 0.38. The low precision of the client-based method is due to the relatively important number of false positives compared to Botometer. However, our simple method shows a good accuracy. This results is not surprising since our method eliminates all tweets from third party Twitter clients which are not necessarily used to make bots.

Very advanced bots might not be detected by our method, but this is also a problem for more advanced methods that rely on a training set of known bots^61^. However, our analysis shows that the number of rejected tweets represents a small fraction of the total number of tweets (≤8%).

### Hashtag classification

We split our dataset in two parts. The first part, from June 1st to September 1st, covers the two conventions and the second part, from September 1st to November 8th, covers the three presidential debates until election day. This allows us verify the consistency of our results and evaluate the quality of our model in a realistic context by training it only on the first part of our dataset and evaluating it on the second part (see Section “Comparison with national polls aggregate”).

We build a labeled training set of tweets with explicit opinion about the two presidential candidates by taking advantage of the fact that a large number of Twitter users label their own tweets by using hashtags. The use of a hashtag that explicitly expresses an opinion in a tweet represents a “cost” in terms of self-exposition by Twitter users^34^ and therefore allows one to select tweets that clearly state support or opposition to the candidates.

Our first task is therefore to classify the hashtags present in our dataset as expressing support or opposition to one of the candidate. For this purpose, we start by identifying the most important hashtags in term of their total number of occurrences and then use the relations between hashtags co-occurring in tweets to discover new hashtags.

Among the top occurring hashtags (shown in Table S2), we identify four hashtags each representing a different category: *#maga* for pro-Trump (*maga* is the abbreviation of the official Trump campaign slogan: *Make America Great Again)*, *#imwithher* for pro-Clinton (the official Clinton campaign slogan), *#nevertrump* for anti-Trump and *#neverhillary* for anti-Clinton.

We then construct the hashtag co-occurrence network *H*(*V*, *E*), where the set of vertices *v*_*i*_ ∈ *V* represents hashtags, and an edge *e*_*ij*_ is drawn between *v*_*i*_ and *v*_*j*_ if they appear together in a tweet. For the period going from June 1st until September 1st, the resulting graph has 83,159 vertices and 589,566 edges.

Following reference^62^, we test the statistical significance of each edge *e*_*ij*_ by computing the probability *p*_*ij*_ (*p*-value of the null hypothesis) to observe the corresponding number of co-occurrences by chance knowing the number of occurrences *c*_*i*_ and *c*_*j*_ of the vertices *v*_*i*_ and *v*_*j*_, and the total number of tweets *N*. We keep only significant edges satisfying *p* < *p*_0_, where *p*_0_ = 10^−6^, effectively filtering out spurious relations between hashtags. Finally, a weight *s*_*ij*_ = log(*p*_0_/*p*_*ij*_) representing the significance of the relation between two hashtags is assigned to each edge. Retaining only significant edges and considering only the largest component of the filtered graph, reduces the graph to 8,299 vertices and 26,429 edges.

Using a method inspired by the method of label propagation^45^, we use the resulting co-occurrence network to discover hashtags that are significantly related to the hashtags initially chosen to represents the different classes. We simplify the hashtag classification problem by considering only two classes: *C*_*C*_ for the hashtags pro-Clinton or anti-Trump and *C*_*T*_ for the hashtags pro-Trump or anti-Clinton. Starting from the initial set of hashtags, we infer the class of their neighbors *v*_*i*_ by verifying the following condition: if

5 $$\sum _{j\in {C}_{C}}{s}_{ij} > \sum _{j\in {C}_{T}}{s}_{ij},$$

*v*_*i*_ is assigned to *C*_*C*_. Similarly, if

6 $$\sum _{j\in {C}_{T}}{s}_{ij} > \sum _{j\in {C}_{C}}{s}_{ij},$$

*v*_*i*_ is assigned to *C*_*T*_. Note that *s*_ij_ = *0* if there is no edge between *i* and *j*.

We then further filter the new hashtags by keeping only hashtags having a number of occurrences ${c}_{i} > r\,\mathop{{\rm{\max }}\,}\limits_{{v}_{j}\in {C}_{k}}{c}_{j}$ where *c*_*i*_ is the number of occurrences of the hashtag associated with vertex *v*_*i*_, *C*_*k*_ is the class to which *v*_*i*_ belong and *r* < 1 is a threshold parameter that we set to *r* = 0.001. Finally, a human validation among the new hashtags is performed to only add hashtags that are direct reference to the candidate, its party or slogans of the candidate and that express an opinion. Table S3, shows example of this manual selection.

This entire process is then repeated adding the newly selected hashtags to each class and propagating the selection to their neighbors. After each iteration we also verify the consistency of the classes by removing hashtags that do no longer satisfy Eqs (5) and (6).

After three iterations of this process, we find a stable set of hashtags represented in Fig. 2 and given in Tables S4 and S5 of the Supplementary Information. Applying a community detection algorithm^45,46^ to the final network found with our method results in four different clusters corresponding to the Pro-Clinton, Anti-Clinton, Pro-Trump and Anti-Trump hashtags as shown in Fig. 2. This shows that our resulting classes are well separated and correspond to the partition of the network maximizing Newman’s modularity. The full set of hashtags is given in the Supplementary Information (Tables S4 and S5).

As a robustness check, we study how the agreement using the full set of tweets compares with that using the initial seed set of hashtags to train the supervised model. Using the final set of hashtags instead of the initial set increases the agreement between the Twitter opinion trend and the NYT national polling average (see Fig. S4 of the Supplementary Information). The improvement of the classification is also revealed by the larger classification scores obtained with the final set of hashtags (see Table 1). For example, when using a window length of 13 days, the Pearson correlation coefficient increases from *r* = 0.90 to *r* = 0.94 and the root-mean-square error decreases from RMSE = 0.40% to RMSE = 0.31%. The classification improvement in *F*_1_-score increases from *F*_1_ = 0.73 to *F*_1_ = 0.81.

To assess the robustness of the manual selection of hashtags, we perform our daily classification of users using as a basis for our training set of hashtags three sets, each with a different random sample containing only 90% of our final sets of hashtags. We find that it only slightly affect the final daily classification of users with a root-mean-square deviation of 2.7% between the ratio of users in each camp using the reduced sets of hashtags and the full set of hashtags (see Fig. S3 of the Supplementary Information). This indicates that our method is robust against significant (10%) variation in the manual selection of hashtags.

### Opinion mining

We build a training set of labeled tweets with two classes: 1) pro-Clinton or anti-Trump and, 2) pro-Trump or anti-Clinton. We discard tweets belonging to the both classes simultaneously to avoid ambiguous tweets. We also remove retweets to avoid duplicates in our training set. We select only tweets that were posted using an official Twitter client in order to discard tweets that might originate from bots and to limit the number of tweets posted from professional accounts. We use a balanced set, with the same number of tweets in each class, totaling 835,808 tweets for the first part of our dataset and 682,508 tweets for the second part. The tweet contents is tokenized to extract a list of words, hashtags, usernames, emoticons and urls. The hashtags used for labeling are striped off from the tweets and the other hashtags are kept as they may contain significant information about the opinion of the tweet. We also keep the urls as features since they usually point to resources determining the opinion of the tweet. Moreover, replacing all urls by the token “URL” (creating an equivalent class) resulted in smaller classification score. We use the presence of unigrams and bigrams as features. We find 3.5 million features for the first part of our dataset and 3.1 million for the second part. The performance of different classifiers is tested (Support Vector Machine, Logistic Regression and modified Huber) with different regularization methods (Ridge Regression, Lasso and Elastic net)^63^. Hyperparameter optimization is performed with a 10-fold cross validation optimizing *F*_1_ score^64^. The 10-fold cross validation is done by first randomly shuffling the training set and then splitting it in 10 equally sized folds. Each fold is then used once as a validation while the 9 remaining folds form the training set. The *F*_1_ score is computed as the average between the value obtained by taking each class as the positive class and averaged over all folds. The best score is obtained with a Logistic Regression classifier with *L*_2_ penalty (Ridge Regression). Classification scores are summarized in Table 1. We then classify users according to the class in which the majority of their tweets are classified. The out-of-sample performance of the classifier, computed using a manually annotated set of 500 random tweets, is also shown in Table 1. Among the 500 annotated tweets, 4.2% tweets were judged as neutral and we were unable to classify 6.4%. We take into account neutral and undetermined tweets as false results in the computation of the accuracy but we do not take them into account in the computation of the other scores. Table 1 shows the classification performance at the tweet level, however we ultimately classify users, based on their tweets, which improves the expected performance for users that post several tweets.

**Table 1:** Best classification score achieved using a Logistic Regression Classifier with *L*_2_ regularization.

	*F*1	AUROC	Accuracy	Precision	Recall
Initial set	0.73	0.81	0.71	0.72	0.73
Final set	0.81	0.89	0.81	0.81	0.81
Final set - out-of-sample	0.79	—	0.72	0.79	0.79

For the training set obtained with the final set of hashtags, classification scores are computed over a 10-fold cross-validation. For the training set obtained with the initial set of hashtags, classification scores are computed on the set of tweets contained in the final set but not used for training the classifier. For *F*_1_, Precision and Recall, the average of the two scores computed by taking each class as the positive class is computed. The out-of-sample scores are computed using a random sample of 500 manually annotated tweets.

Although supervised methods that directly estimate the aggregated repartition of opinion have often a better accuracy than methods that classify at the document level^34,37,39^, they do it at the cost of losing the individual classification. Furthermore, these methods estimate the proportion of documents at the aggregated level (in our case tweets) in favor of each candidate and not the proportion of users. This can be problematic when users from different parties tweet, in average, at different rates, see Fig. 6. Here, we extract the opinion of each individual tweet towards a candidate from where we directly extract the opinion of each user, which, in turn, can be used to obtain the percentage of users favoring each candidate.

Using the final set of hashtags instead of the initial set, consisting of the top hashtags in each category, increases the agreement between the Twitter opinion trend and the NYT national polling average (see Fig. S4 of the Supplementary Information). The improvement of the classification is also revealed by the larger classification scores obtained with the final set of hashtags (see Table 1).

### Fit of the Twitter opinion with the National Polling Average

Since ${r}_{w}^{C}(i)=1-{r}_{w}^{T}(i)$ where *C* stand for Clinton and *T* for Trump in eq. 1, we only fit ${r}_{w}^{C}(i)$ with its NYT counterpart *y*^*C*^(*i*). We have the following relations between the optimal parameters between the Clinton fit and the Trump fit: *A*^*T*^ = *A*^*C*^ and *b*^*T*^ = 1 − *b*^*C*^ − *A*^*C*^, since *y*^*C*^(*i*) = 1 − *y*^*T*^(*i*). We omit the superscript *k* in the following.

The backward window average at day *i* of length *w* days of the support ratio *r* is defined as

7 $${r}_{w}(i)=\frac{1}{w}\sum _{j=i-w+1}^{i}r(j)$$

where *r*(*j*) is the ratio of users in favor of a candidate at day *j*. The moving window average converts fluctuating daily data into a smooth trend that can be compared with the NYT smooth time series aggregated over many polls performed over several days. We fit the values of *A*, *b* and *t*_*d*_ for increasing values of *w* by minimizing the mean squared error.

Since a backward moving average induces a backward time shift, the total forward time shift between the Twitter time series and the NYT polling average is given by ${T}_{d}={t}_{d}+\frac{w-1}{2}$, where we limit *w* to odd positive integer values to have only integer values.

### Data and materials availability

The datasets analysed during the current study are available from the corresponding author on reasonable request.

## Electronic supplementary material

Supplementary Information
