# Supplementary material for: Validation of Twitter opinion trends with national polling aggregates: Hillary Clinton vs Donald Trump
Source: Sci Rep. 2018 Jun 6;8:8673. doi: 10.1038/s41598-018-26951-y (PMC5989214; doi:10.1038/s41598-018-26951-y)
Supplement: Supplementary file 1 — Supplementary Information [file 41598_2018_26951_MOESM1_ESM.pdf]

Validation of Twitter opinion trends with national polling aggregates: Hillary Clinton vs Donald Trump Supplementary Information

Alexandre Bovet[1], Flaviano Morone[1], and Hernán A. Makse[1,*]

[1]Levich Institute and Physics Department, City College of New York, New York, New York 10031, USA
[*]hmakse@lev.ccny.cuny.edu

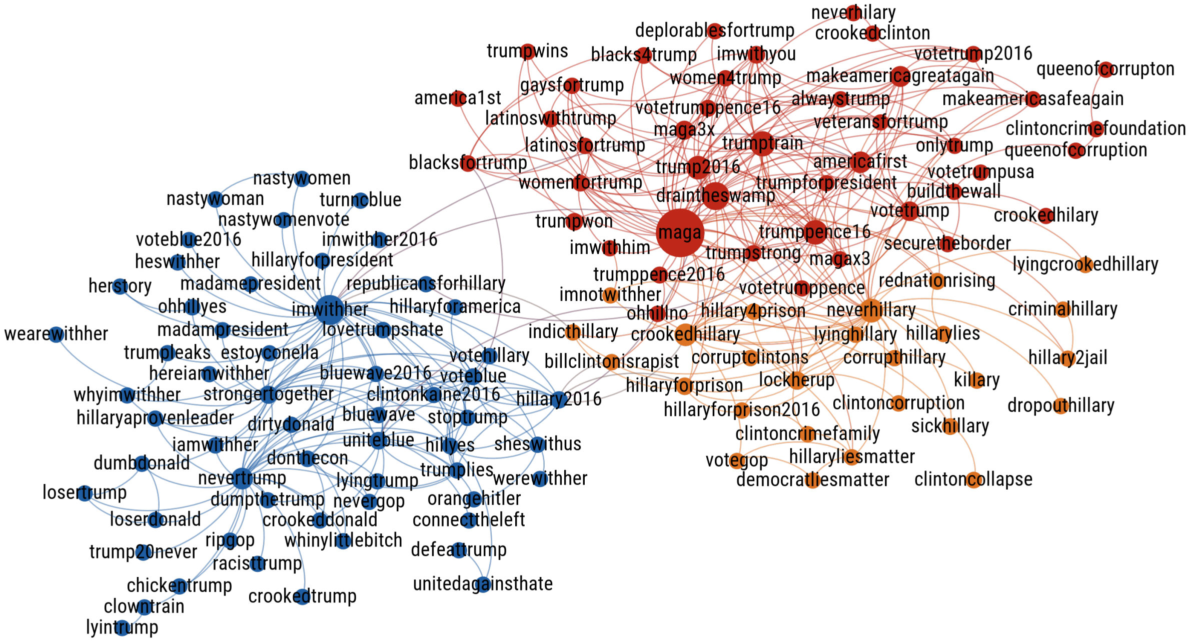

Figure S1. Hashtag classification via network of co-occurrence for September 1st to November 8th. Network of hashtags obtained by our algorithm from September 1st to November 8th. Nodes of the network represent hashtags and an edge is drawn between two hashtags when their co-occurrence in tweets is significant. The size of the node is proportional to the total number of occurrence of the hashtag. Similarly to the network for June 1st to September 1st (Fig. 2 in the main paper), two main clusters are visible, corresponding to the Pro-Trump/Anti-Clinton and Pro-Clinton/Anti-Trump hashtags. Inside of these two clusters, the separation between Pro-Trump (red) and Anti-Clinton (orange) is visible and the Pro-Clinton and Anti-Trump form a single cluster (blue). The coloring corresponds to clusters found by community detection.

client name	number of tweets
Twitter for iPhone	60 119 804
Twitter for Android	39 391 345
Twitter Web Client	36 416 726
Twitter for iPad	11 563 812
Mobile Web (M5)	3 680 822
TweetDeck	2 310 956
Facebook	1 228 664
Twitter for Windows	690 012
Mobile Web (M2)	629 098
Twitter for Windows Phone	540 218
Mobile Web	442 489
Google	403 207
Twitter for BlackBerry	279 754
Twitter for Android Tablets	186 652
Twitter for Mac	158 318
iOS	101 666
Twitter for BlackBerry®	68 379

Table S1. List of Twitter official clients. We also display the number of tweets originating from each official client. The number of tweets originating from official clients represent 92% of the total number of tweets.

Hashtag	Number of occurrences
trump	2 240 499
trump2016	1 320 217
maga	1 139 644
hillary	905 065
hillaryclinton	718 159
imwithher	690 519
trumptrain	654 573
neverhillary	634 562
demsinphilly	627 446
nevertrump	560 876
tcot	531 389
rncincle	498 718
trumppence16	473 924
donaldtrump	409 708
crookedhillary	396 836

Table S2. Top occurring hashtags from June 1st to September 1st 2016.

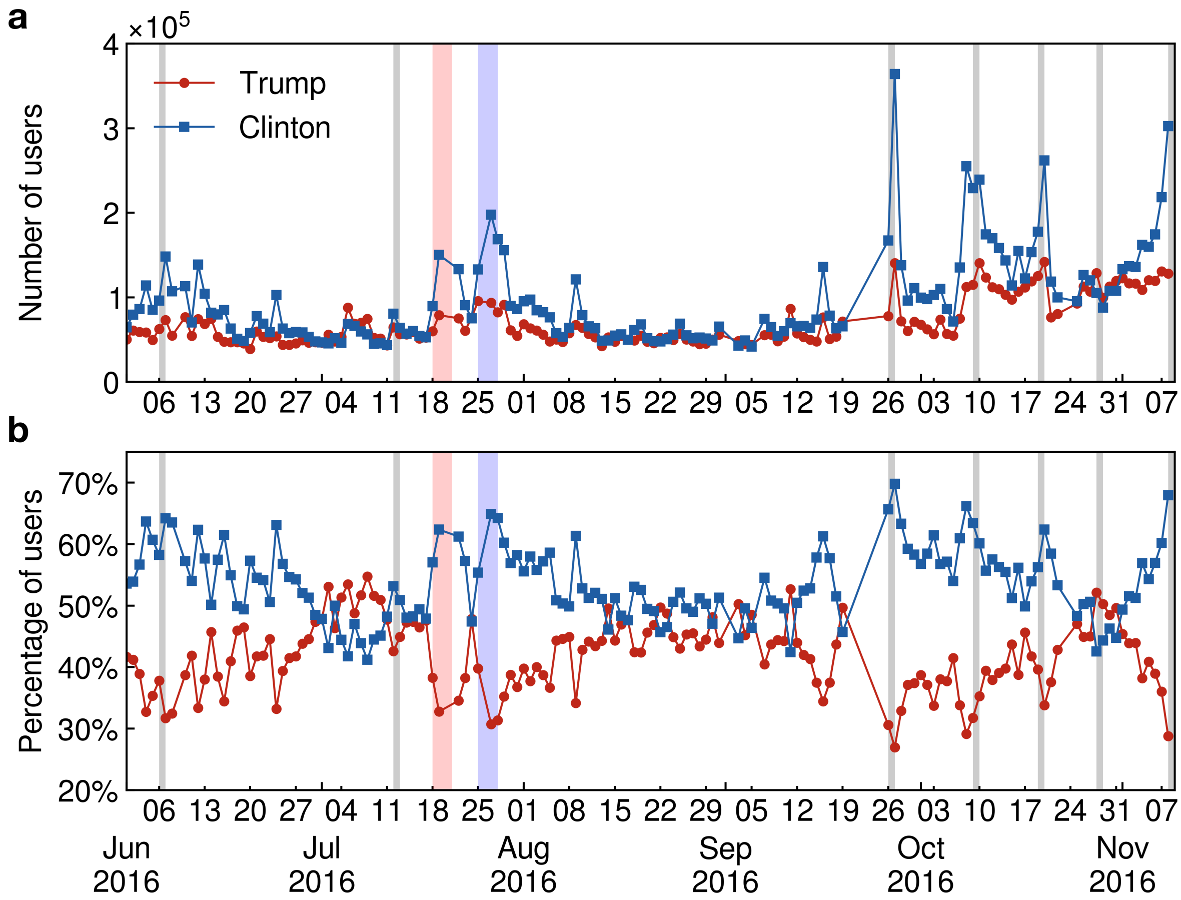

Figure S2. Supporters in the filtered Twitter dataset. (**a**) Absolute number and (**b**) percentage of users labeled as Trump (red) and as Clinton (blue) in the filtered dataset as a function of time. To asses the importance of the possible noise in the data induced by the popular "trump" and "hillary" keywords, we filter our dataset to keep only tweets with either one of the following keywords : 'realdonaldtrump', 'hillaryclinton', 'donaldtrump' or at least one of the following pairs of keywords 'trump' and 'donald' or 'hillary' and 'clinton'. Although this keyword filtering reduces the dataset from 158 millions tweets to 58 millions tweets (considering only tweets from official clients), our results are not significantly changed. The relative number of each supporters and the temporal evolution of the number of users is similar to the results obtained from our full dataset.

Associated with C_C	Added to C_C	Associated with C_T	Added to C_T
vote	strongertogether	radicalislam	trumptrain
republican	donthecon	ccot	trumppence16
america	voteblue	corruption	votetrump
hillaryclinton	dumptrump	ryan	hillno
real	hillary2016	fbi	handcuffhillary
racist	uniteblue	hillary	imnotwithher
p2	clintonkaine2016	tcot	votetrump2016
veep	hillyes	jobs	crookedhillary
trumpuniversity	nevertrumppence	tcot	hillaryforprison
kkk	chickentrump	scotus	maga

Table S3. Example of hashtags discovered in the co-occurrence network. We show hashtags associated with each class but not selected and hashtags selected as they directly reference one of the candidate or its party and express an opinion. The list of hashtags associated with each candidate shows how the hashtag co-occurrence network can be used discover the topics commonly discussed by supporters of each camps.

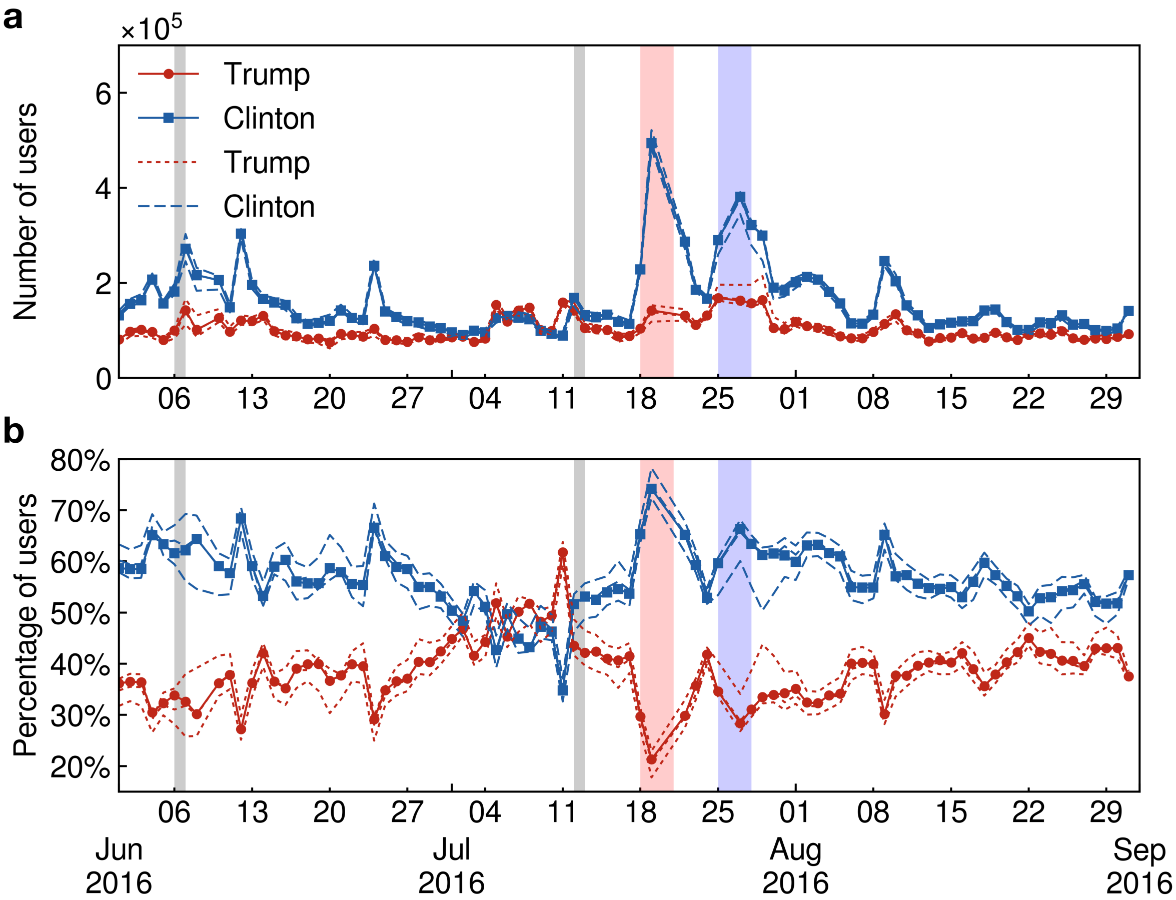

Figure S3. Robustness test of the hashtag selection. Comparison of absolute number of daily users (**a**) and percentage of users (**b**) using the full final set of hashtags (continuous lines) with results using three random subsets with 90% of the final set of hashtags (dotted and dashed lines) for the period from June 1st to September 1st. The root-mean-square deviation between the daily percentage of users found with the full set of hashtags and with the reduced set is RMSD = 2.7%. These results show that our method is robust against variation in the manual section of hashtags used to build the training set of tweets.

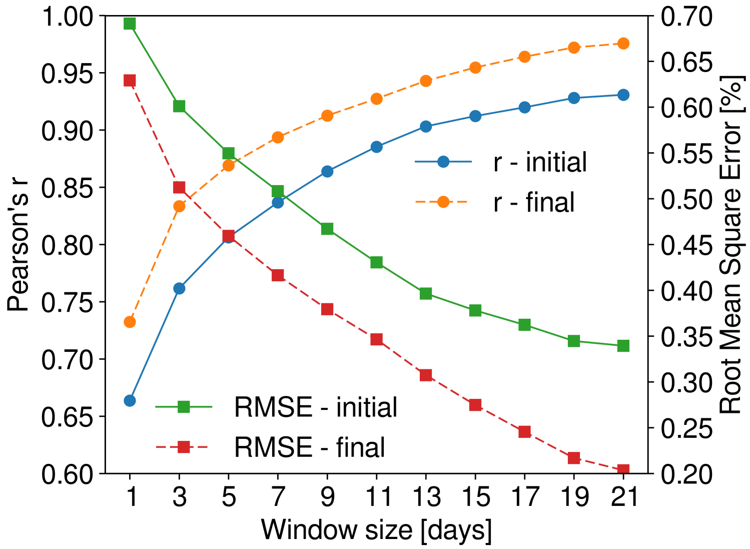

Figure S4. Improvement of the quality of fit when using the final set of hashtags compared to the initial set. Using the final set of hashtags for building our training set instead of the initial set improve the agreement between our Twitter opinion trend and the NYT Polling Average. This is shown by a larger Pearson's correlation coefficient (r, circles) and a lower root-mean-square error (RMSE, squares). When using a window length of 13 days, the Pearson correlation coefficient increases from $r = 0.90$ to $r = 0.94$ and the root-mean-square error decreases from RMSE = 0.40% to RMSE = 0.31%.

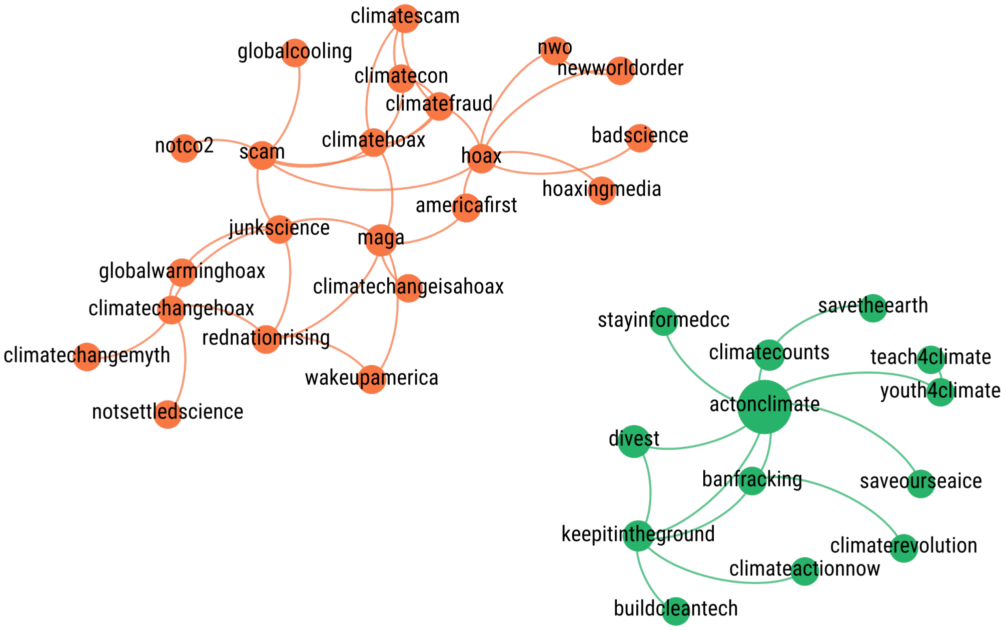

Figure S5. Hashtag co-occurrence networks for climate change. The network splits into two groups, one with hashtags supporting action toward climate change (green) and the other with hashtags depicting climate change as a hoax (orange). This result suggests that our machine learning and co-occurrence hashtag network method can be generalized to topics beyond the election setting.

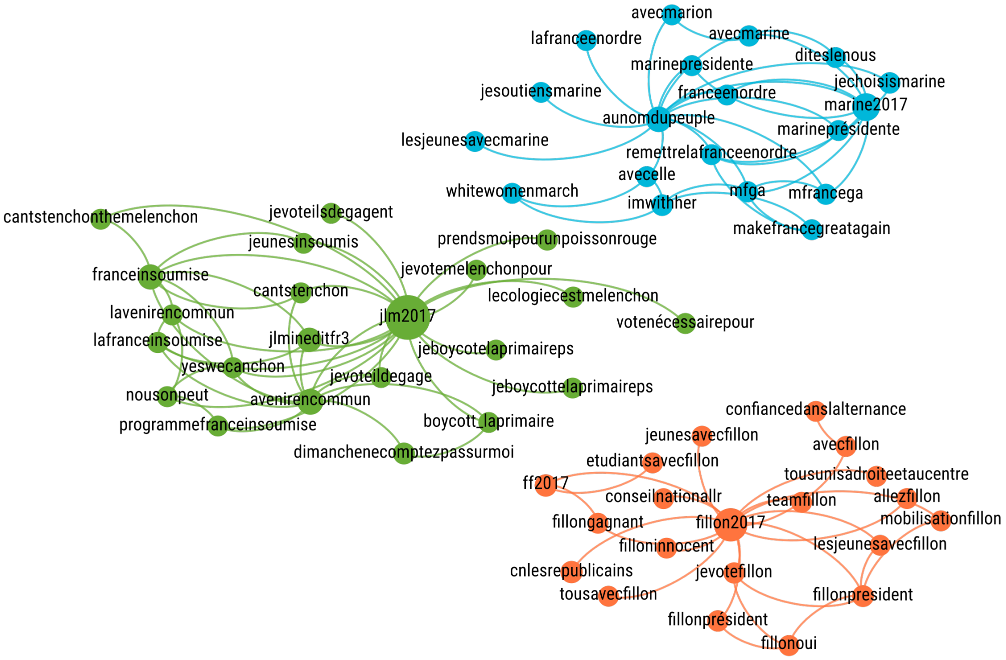

Figure S6. Hashtag co-occurrence networks for three candidates of the French presidential elections. A separation of the co-occurrence network into three clear clusters is achieved for the hashtags employed by users expressing supports to three candidates to the French presidential election (François Fillon (orange), Marine Le Pen (blue) and Jean-Luc Mélanchon (green)). They correspond to the three main confirmed candidates at the time of data acquisition (December 19th, 2016 to January 31st, 2017). This is the reason why we did not acquire data about Emmanuel Macron, who ultimately passed the first round of the elections along with Marine Le Pen. Each group expresses predilection for each of the three French presidential candidates.

pro-Clinton	anti-Trump	pro-Trump	anti-Clinton
bernwithher	antitrump	alwaystrump	clintoncorruption
bluewave2016	anyonebuttrump	babesfortrump	clintoncrime
clintonkaine2016	boycotttrump	bikers4trump	clintoncrimefamily
estoyconella	chickentrump	bikersfortrump	clintoncrimefoundation
herstory	clowntrain	blacks4trump	corrupthillary
heswithher	crookeddonald	buildthatwall	criminalhillary
hillafornia	crookeddrumpf	buildthewall	crookedclinton
hillary2016	crookedtrump	cafortrump	crookedclintons
hillaryforamerica	crybabytrump	democrats4trump	crookedhilary
hillaryforpr	defeattrump	donaldtrumpforpresident	crookedhiliary
hillaryforpresident	dirtydonald	feelthetrump	crookedhillary
hillarysopresidential	donthecon	feminineamerica4trump	crookedhillaryclinton
hillarysoqualified	drumpf	gays4trump	deletehillary
hillarystrong	dumbdonald	gaysfortrump	dropouthillary
hillstorm2016	dumpthetrump	gotrump	fbimwithher
hillyes	dumptrump	heswithus	handcuffhillary
hrc2016	freethedelegates	imwithhim	heartlesshillary
hrcisournominee	lgbthatestrumpparty	imwithyou	hillary2jail
iamwithher	loserdonald	latinos4trump	hillary4jail
imwither	losertrump	latinosfortrump	hillary4prison
imwithher	lovetrumpshate	maga	hillary4prison2016
imwithher2016	lovetrumpshates	makeamericagreat	hillaryforprison
imwithhillary	lyindonald	makeamericagreatagain	hillaryforprison2016
imwiththem	lyingdonald	makeamericasafeagain	hillaryliedpeopledied
itrusther	lyingtrump	makeamericaworkagain	hillarylies
itrusthillary	lyintrump	onlytrump	hillaryliesmatter
madamepresident	makedonalddrumpfagain	presidenttrump	hillarylostme
madampresident	nevergop	rednationrising	hillaryrottenclinton
momsdemandhillary	nevertrump	trump16	hillarysolympics
ohhillyes	nevertrumppence	trump2016	hillno
readyforhillary	nodonaldtrump	trumpcares	hypocritehillary
republicans4hillary	notrump	trumpforpresident	imnotwithher
republicansforhillary	notrumpanytime	trumpiswithyou	indicthillary
sheswithus	poordonald	trumppence16	iwillneverstandwithher
standwithmadampotus	racisttrump	trumppence2016	killary
strongertogether	releasethereturns	trumpstrong	lockherup
uniteblue	releaseyourtaxes	trumptrain	lyingcrookedhillary
vote4hillary	ripgop	veteransfortrump	lyinghillary
voteblue	showusyourtaxes	vets4trump	lyinhillary
voteblue2016	sleazydonald	votegop	moretrustedthanhillary
votehillary	stoptrump	votetrump	neverclinton
welovehillary	stupidtrump	votetrump2016	nevereverhillary
yeswekaine	traitortrump	votetrumppence2016	neverhillary
	treasonoustrump	woman4trump	neverhilllary
	trump20never	women4trump	nohillary2016
	trumplies	womenfortrump	nomoreclintons
	trumpliesmatter		notwithher
	trumpsopoor		ohhillno
	trumpthefraud		releasethetranscripts
	trumptrainwreck		riskyhillary
	trumptreason		shelies
	unfittrump		sickhillary
	weakdonald		stophillary
	wherertrumpstaxes		stophillary2016
	wheresyourtaxes		theclintoncontamination
	whinylittlebitch		wehatehillary
	womentrumpdonald		whatmakeshillaryshortcircuit

Table S4. List of hashtags used for labeling the tweet training set from June 1st to September 1st.

pro-Clinton	anti-Trump	pro-Trump	anti-Clinton
bluewave	chickentrump	alwaystrump	billclintonisrapist
bluewave2016	clowntrain	america1st	clintoncollapse
clintonkaine2016	crookeddonald	americafirst	clintoncorruption
connecttheleft	crookedtrump	blacks4trump	clintoncrimefamily
estoyconella	defeattrump	blacksfortrump	clintoncrimefoundation
hereiamwithher	dirtydonald	buildthewall	corruptclintons
herstory	donthecon	deplorablesfortrump	corrupthillary
heswithher	dumbdonald	draintheswamp	criminalhillary
hillary2016	dumpthetrump	gaysfortrump	crookedclinton
hillaryaprovenleader	loserdonald	imwithhim	crookedhilary
hillaryforamerica	losertrump	imwithyou	crookedhillary
hillaryforpresident	lovetrumpshate	latinosfortrump	democratliesmatter
hillyes	lyingtrump	latinoswithtrump	dropouthillary
iamwithher	lyintrump	maga	hillary2jail
imwithher	nastywoman	maga3x	hillary4prison
imwithher2016	nastywomen	magax3	hillaryforprison
madamepresident	nastywomenvote	makeamericagreatagain	hillaryforprison2016
madampresident	nevergop	makeamericasafeagain	hillarylies
ohhillyes	nevertrump	onlytrump	hillaryliesmatter
republicansforhillary	orangehitler	rednationrising	imnotwithher
sheswithus	racisttrump	securetheborder	indicthillary
strongertogether	ripgop	trump2016	killary
turnncblue	stoptrump	trumpforpresident	lockherup
uniteblue	trump20never	trumppence16	lyingcrookedhillary
unitedagainsthate	trumpleaks	trumppence2016	lyinghillary
voteblue	trumplies	trumpstrong	neverhilary
voteblue2016	whinylittlebitch	trumptrain	neverhillary
votehillary		trumpwins	ohhillno
wearewithher		trumpwon	queenofcorruption
werewithher		veteransfortrump	queenofcorrupton
whyimwithher		votegop	sickhillary
		votetrump	
		votetrump2016	
		votetrumppence	
		votetrumppence16	
		votetrumpusa	
		women4trump	
		womenfortrump	

Table S5. List of hashtags used for labeling the tweet training set from September 1st to November 8th.
